# Understanding antibiotic decision-making for bovine respiratory disease: a survey of UK farm veterinarians

**DOI:** 10.3389/fvets.2026.1791479

**Published:** 2026-05-19

**Authors:** Ardi B. Prakoso, Gwenllian M. Rees

**Affiliations:** 1 Department of Life Sciences, Aberystwyth University, Aberystwyth, Ceredigion, United Kingdom; 2 Department of Life Sciences, School of Veterinary Science, Aberystwyth University, Aberystwyth, Ceredigion, United Kingdom

**Keywords:** antibiotic prescribing behaviour, antimicrobial stewardship, bovine respiratory disease, diagnostic and preventive practices, veterinarian-farmer relationships

## Abstract

**Introduction:**

Bovine Respiratory Disease (BRD) is a major cause of morbidity and mortality in young calves and a major driver of antibiotic use in the United Kingdom (UK), making veterinary decision-making central to antimicrobial stewardship (AMS).

**Methods:**

This study used a mixed-methods survey of UK farm veterinarians (*n* = 95), combining quantitative and qualitative questions, to explore diagnostic tools, antibiotic choices, and factors influencing the use of ancillary therapies.

**Results:**

For BRD diagnosis in calves, clinical examination (36.8%), necropsy (33.7%), and rectal temperature (31.6%) were the tools most regarded as extremely important. Oxytetracycline was the most commonly used primary antibiotic (52.6%), tulathromycin was the secondary option (38.9%), and NSAIDs were represented as the most common ancillary therapy (80.0%). The risk of antimicrobial resistance (AMR; 22.1%) and clinical experience (21.1%) primarily drove antibiotic selection. Prevention focused on colostrum management (80.0%), ventilation (65.3%), intranasal vaccines (46.3%), and injectable vaccines (40.0%). Influences on prescribing behaviours included motivations regarding responsible antibiotic use (68.4%), avoiding blame (45.3%), and veterinarian-farmer relationships (42.1%). Bacterial culture and sensitivity testing were the least important (24.2%), suggesting a need to strengthen AMS.

**Discussion:**

These results showed a phenomenon in clinical diagnosis, specific antimicrobial classes used, and relational factors in prescribing. The study showed that clinical assessment, social pressures, and policy contexts jointly shaped antimicrobial prescribing decisions. Enhancing diagnostic capacity, embedding evidence-based preventive measures, and applying behavioural frameworks to support veterinarian-farmer communication were identified as essential to improve AMS. The targeted interventions within One Health approach are required to support sustainable UK dairy health management.

## 1 Introduction

Bovine Respiratory Disease (BRD) is among the most significant economic and clinical health challenges affecting calves during the first 6 months of life, involving both the upper and lower respiratory tracts (1, 2). Etiologically, BRD is a multifactorial disease arising from interactions between host susceptibility and multiple pathogens. Primary viral agents, such as bovine respiratory syncytial virus (BRSV), predispose cattle to secondary bacterial infections by *Mannheimia haemolytica* and *Pasteurella multocida*, causing severe bronchopneumonia. Accordingly, BRD control measures combine prevention, such as vaccination and herd-management strategies, with treatment, which primarily relies on antimicrobial use (3, 4).

Previous research reported that the disease affects 12–16% of preweaned dairy calves globally, accounting for approximately one-quarter of calf mortalities (3, 5, 6). Besides acute morbidity and mortality, BRD has long-term consequences into adulthood, including reduced growth rates, lower milk yield, and an increased risk of culling before the initial lactation period is completed (7, 8). In addition to productivity losses, BRD is a painful condition in calves, characterised by behavioural and physiological indicators, such as reduced feed intake, prolonged lying period, and elevated inflammatory biomarkers (9, 10).

The economic burden is considerable, comprising direct costs, namely veterinary treatment and mortality, as well as indirect losses including reduced weight gain, prolonged time to market, and decreased reproductive performance (11). In the United Kingdom (UK), the cost per affected calf is estimated at between £30 and £80, with industry-wide impacts totalling around £50 million annually (12). These findings reinforce BRD’s status as one of the most frequent causes of veterinary intervention in dairy cattle, highlighting its central role in UK livestock health management (6). The disease also accounts for a substantial proportion of antibiotic use in calves and is regarded as a priority for antimicrobial stewardship (AMS) in UK dairy farm production systems (13–15). However, there is limited knowledge about UK farm veterinarians’ decision-making regarding the use of antibiotics in the treatment of BRD.

Antimicrobial resistance (AMR) is a complex, transboundary challenge that endangers human, animal, and environmental health systems. Addressing this global issue demands a collaborative One Health approach that integrates cross-sector expertise and interventions (16–18). In this context, veterinarians’ antibiotic prescribing practices intersect with broader AMR concerns, connecting farm-level decision-making to global health priorities. The interconnected nature of AMR, human, and animal health reflects the importance of interdisciplinary approaches that combine clinical, epidemiological, and behavioural sciences (19).

While antibiotics, particularly oxytetracycline, tulathromycin, and florfenicol, remain central to BRD control, their use is increasingly scrutinised due to AMR risks (20–22). To address these concerns, the European Medicines Agency (EMA) classifies veterinary antibiotics into 4 groups (A-D) based on their public health importance to guide prudent antibiotic use. Category D (‘Prudence’) serves as the primary line of treatment, whereas Category B (‘Restrict’) is restricted to treating infections only where no alternatives are available (23). In the UK, policy frameworks such as Confronting Antimicrobial Resistance 2024 to 2029 focus on reducing unnecessary antimicrobial use through diagnostic stewardship, sensitivity testing, and evidence-based prescribing (24). However, a UK survey found that fewer than one-fifth (19%) of farm veterinarians performed antimicrobial susceptibility testing (AST) when a bacterial infection was first suspected (25). Barriers to the responsible use of antibiotics include cost, time constraints, a lack of rapid diagnostic tools, and farmer expectations (25, 26).

International prescribing practices reveal varying approaches to BRD treatment, though macrolides and tetracyclines remain among the most commonly used antibiotic classes across regions. In the United States (US), cross-sectional surveys of veterinarians managing BRD in preweaned dairy calves showed widespread use of antibiotics as first-line treatment, most commonly tulathromycin, with florfenicol as a frequent second-line choice (9). Similarly, in Australian feedlots, national guidelines recommended tetracycline and tilmicosin as first-line agents, though severe cases may progress to tulathromycin or ceftiofur (27). Clinical trials conducted across Europe further support the efficacy of antibiotics such as tulathromycin in managing predominant bacterial infections, including *M. haemolytica* and *P. multocida* (28).

In the UK context, research has focused primarily on producer practices and broad prescribing factors. Surveys of Scottish dairy farmers managing calves have reported macrolides and tetracyclines as the most commonly used antibiotics for calf pneumonia (29). Complementary insights into veterinary prescribing behaviour across the UK indicate that decisions are shaped by a combination of clinical factors (e.g., pyrexia), practical considerations (e.g., ease of administration and withdrawal periods), and external commercial pressures (e.g., supermarket contracts and assurance schemes) (30). Furthermore, ethnographic case studies highlight how AMS pressures and concerns over treatment efficacy can influence farm-level management practices, such as shifting calf housing systems to reduce disease incidence (31). However, despite the global data and UK contextual studies, none of these UK studies directly examined antibiotic usage for BRD from the specific perspective of UK farm veterinarians, leaving a significant knowledge gap regarding the factors that influence their prescribing decisions and the evidence base for BRD treatment strategies. To address this gap, the present research examines the decision-making processes and contextual factors that influence antibiotic prescribing for BRD in calves among UK farm veterinarians.

## 2 Materials and methods

The study was approved by the Research Ethics Institutional Process at Aberystwyth University (application number: 29490Ha; May 2025).

### 2.1 Survey

The survey design was based on research by Mijares et al. (9), which investigated farm veterinarians’ perspectives on treatment and diagnostic tools related to BRD in preweaned calves in the US. The survey questions were adapted for the UK context, incorporating additional questions from relevant UK-based research by Coyne et al. (25).

While the original US survey drew upon either the Wisconsin or California Respiratory Scoring Systems, this modified UK survey focuses on common clinical signs (e.g., cough, dyspnoea, and nasal discharge). Also, specific antimicrobials referenced within the survey were modified - the US-specific antibiotic trade name and active ingredients being replaced with commercial products licensed and commonly used in the UK market, with classification aligned with the National Office of Animal Health (NOAH) Compendium of Animal Medicines website (32).

The paper-based survey was designed using Microsoft Word (Microsoft Corporation, Redmond, WA). It was piloted with farm veterinarians (*n* = 27) during the Arwain DGC conference held in May 2025. Feedback gathered during this pilot study was used to assess the clarity of the phrasing, the clinical relevance of the scenarios, and the appropriate sequencing of questions. Minor amendments were required, and the survey was refined, formatted, and advanced in line with the initial feedback.

The final survey comprised a total of 28 questions, including a combination of multiple-choice, text-entry, Likert-scale (33), and numerical rank-order problems (1 to 3) for the first, second, and third choices of antibiotic treatment options in each scenario. Meanwhile, for the Likert-scale questions, a 5-point scale assessed importance, ranging from not at all, slightly, moderately, very, and extremely. The 3-point scale questions were adapted from the UK prior research study by Coyne et al. (25), with the following options: less likely, neutral, and more likely, never or rarely, sometimes, and often or always for the different scenarios and circumstances.

The survey was divided into four main sections: demographics; clinical signs and diagnostic tools; treatment and control measures; and other factors influencing the decision to prescribe an antibiotic (see Supplementary material). The definition of BRD for the purposes of the survey was outlined in the introduction as an infectious disease affecting the upper and lower respiratory tracts of cattle less than 6 months old.

### 2.2 Respondents and survey dissemination

The target population for this study was UK farm veterinarians with working experience treating BRD in calves on dairy cattle farms. While there are 31,279 UK Practising Veterinary Surgeons registered with the Royal College of Veterinary Surgeons (RCVS), the relevant population for this study were the 1,760 individuals stating “farm animal” as their primary species type. The accessible sampling frame was further constrained to the 575 farm animal practitioners who had consented to their data being used for research purposes, only a proportion of whom would be expected to be treating cattle. The final valid sample size achieved (*n* = 95) represented approximately 16.5% of this consented population. Given the exploratory and descriptive nature of this cross-sectional study, the sample size was determined to maximise the proportion of the constrained, accessible population.

The survey was disseminated in paper format during two veterinary-related events in Wales in May and July 2025. The online survey was disseminated via the official research mailing list of veterinarians who identified as UK farm-practising with the RCVS, as well as through the British and Irish Society of Animal Science (BISAS) mailing list and the newsletter of the British Cattle Veterinary Association (BCVA). It was also promoted on social media platforms. Respondent participation was voluntary and anonymous, with a reminder email and post uploaded approximately 14 days after the first broadcast messages.

The survey was live for 3 months between May and August 2025; a total of 95 veterinarians responded.

### 2.3 Statistical analysis

Data from both the paper and online surveys were recorded and extracted from Joint Information Systems Committee (JISC), and subsequently imported into Microsoft Excel (Microsoft Corporation, Redmond, WA) for initial data processing. Data cleaning involved removing incomplete responses. Surveys with an item completion rate of less than 80% (i.e., those with greater than 20% item non-response) were excluded from the final analysis. This stringent requirement was applied to mitigate partial non-response bias, resulting in a more accurate representation of the data. Removing cases with significant non-response was a commonly used approach for handling respondent breakoff in survey research (34). Upon applying this criterion, all 95 submitted responses successfully met the 80% completion threshold. Consequently, no responses had to be excluded, preserving the final analytical sample size at *n* = 95. The remaining results were categorised using descriptive statistics and reported as frequencies and percentages (n, %). Meanwhile, Likert-scale data were summarised as category proportions and represented using stacked bar charts. Potential association between veterinarians demographics (such as age, gender, and working experience) and diagnostic methods were initially analysed using a Chi-square test. For variables with a significant overall association, Fisher’s Exact Test was used in *post-hoc* binary analyses to calculate the Odds Ratio (OR). All statistical analyses were performed using R statistical software (R Foundation for Statistical Computing, Vienna, Austria). A *p*-value of < 0.05 was considered to indicate statistical significance.

### 2.4 Qualitative analysis

Free-text responses were analysed qualitatively using an inductive coding approach, following the procedure described by Maguire and Delahunt (35). Responses were coded and categorised by topic. In addition, illustrative quotes from these responses were used to expand on the quantitative data and to explore further the respondents’ decision-making regarding diagnostic tools, first-line antibiotic treatment, ancillary therapies, and control measures.

## 3 Results

The survey received 95 responses from UK farm-practising veterinarians. Most respondents were aged 35 to 44 years (*n* = 39, 41.1%), followed by those 25 to 34 (*n* = 29, 30.5%), 45 to 54 (*n* = 14, 14.7%), 55 to 64 (*n* = 10, 10.5%), and both under 25 and 65 to 74 (*n* = 1, 1.1% each). Female respondents comprised the majority, making up 56 (58.9%) of the sample. The largest professional experience group had over 20 years in practice (*n* = 23, 24.2%). Geographically, most were based in England (*n* = 44, 46.3%) and Wales (*n* = 41, 43.2%). The largest proportion treated relatively few cattle: 41 (43.2%) treated 50–74% of their cattle, and 50 (52.6%) were active members of the Veterinary Prescribing Champions Network.

### 3.1 Diagnosis

The clinical signs frequently rated as extremely important for diagnosing BRD in calves were difficulty in breathing or dyspnoea (*n* = 56, 58.9%), open-mouth breathing (*n* = 45, 47.4%), fever or pyrexia (*n* = 42, 44.2%), and increased respiratory rate (*n* = 38, 40.0%). In contrast, nasal (*n* = 42, 44.2%) and ocular discharges (*n* = 37, 38.9%) were perceived as moderately important, while poor body condition was categorised as “not at all” rating (*n* = 11, 11.6%).

Beyond clinical signs, veterinarians reported using a range of diagnostic tools. Rectal temperature or fever tags were rated highly, with rectal temperature specifically rated as very important by 53 (55.8%) respondents and as extremely important by 30 (31.6%) respondents. Clinical examination, including auscultation, was also rated highly, with 35 (36.8%) of veterinarians considering it extremely important. Post-mortem examination of deceased animals was similarly rated, with 32 (33.7%) of veterinarians considering it extremely important.

By contrast, imaging methods such as ultrasound and X-ray were ranked as less important, with about one-third (*n* = 31, 32.6%) of veterinarians reporting they were not important. Nevertheless, as shown in Table 1 and supported by representative quotes, these imaging tools found utility in specific situations, such as proactive herd management. Additionally, qualitative responses highlighted alterations in eating habits as another helpful sign, particularly for assessing disease duration.

**Table 1:** Summary of topics regarding the question “Please explain your answer further regarding the importance of these diagnostic tools of BRD in calves” (*n* = 44 responses).

Topic	Definition	Quote
Laboratory diagnostic testing, including bacterial culture, blood sampling, PCR, and sensitivity testing	A suite of analytical procedures useful for characterising, identifying, and detecting pathogens.	*“My approach when BRD is gathering samples to be send to external lab to target the correct treatment.”* *“Nasal PCR and paired antibody ELISA testing are my main tools for this.”*
Clinical examination, including auscultation	A structured physical assessment of an animal’s health status to identify signs of disease or abnormality, involving observation, palpation, percussion, and auscultation.	*“Clinical exam is diagnostic tool used most frequently!!”* *“Observation of breathing pattern often more helpful than auscultation but I do both together.”*
Post-mortem examination for deceased animals	A systematic dissection and analysis of an animal’s body to determine the cause of death.	*“PM is the most useful.”* *“Lab and pm tests are very useful for outbreaks and ongoing problems.”*
Rectal temperature or fever tag	A body temperature monitor can be applied rectally or attached to the ear.	*“Rectal temperature is a quantitative easily measurable and cheap.”* *“Bare minimum is rectal temp and auscultation.”*
Imaging techniques, including ultrasound and X-ray	A couple of non-invasive methods to visualise internal anatomical structures for assessing physiological conditions.	*“Transthoracic ultrasound of calves is used in our practice to screen cohorts on holdings which are engaged in proactive management of BRD.”* *“We have started to use thoracic ultrasound as a prognostic indicator once BRD is diagnosed on farm.”*
Alterations in eating behaviour	Reduced appetite.	*“Monitoring animal behaviour,* e.g.*, feeding patterns and activity is more suggestive of duration of problem and is readily available information to access versus ultrasound etc.”*

Further analysis evaluated if demographic factors shaped specific diagnostic tool preferences. The data showed no statistically significant association between younger or less experienced veterinarians and a higher reliance on advanced technologies like ultrasound (*p* > 0.05). Conversely, standard diagnostic methods, such as clinical examination and auscultation, demonstrated an initial gender-based association (*p* = 0.013). However, a subsequent binary analysis revealed no significant difference between male and female veterinarians (OR = 0.94; *p* = 1.00). Although the Odds Ratio (OR) indicates a marginal tendency for male veterinarians to rate the importance higher than their female counterparts, this difference lacked statistical significance. Consequently, the initial overall significance was primarily driven by the distinct responses within the “other” gender category, rather than a distinct divergence between male and female practitioners.

Similarly, the veterinarian’s years of experience demonstrated an initial significant overall impact on their valuation of clinical examinations (*p* = 0.015). Nevertheless, when specifically comparing veterinarians with less than 6 years of experience to those with six or more years, the *post-hoc* OR indicated no statistically significant difference between these specific cohorts (OR = 0.835; *p* = 0.725). While the vast majority of respondents, regardless of their experience level, tended to rate clinical examinations as “very important” or “extremely important,” neither increasing experience nor binary gender differences were the primary drivers of this valuation.

### 3.2 Treatments

#### 3.2.1 Antibiotic therapies

**Figure 1:**
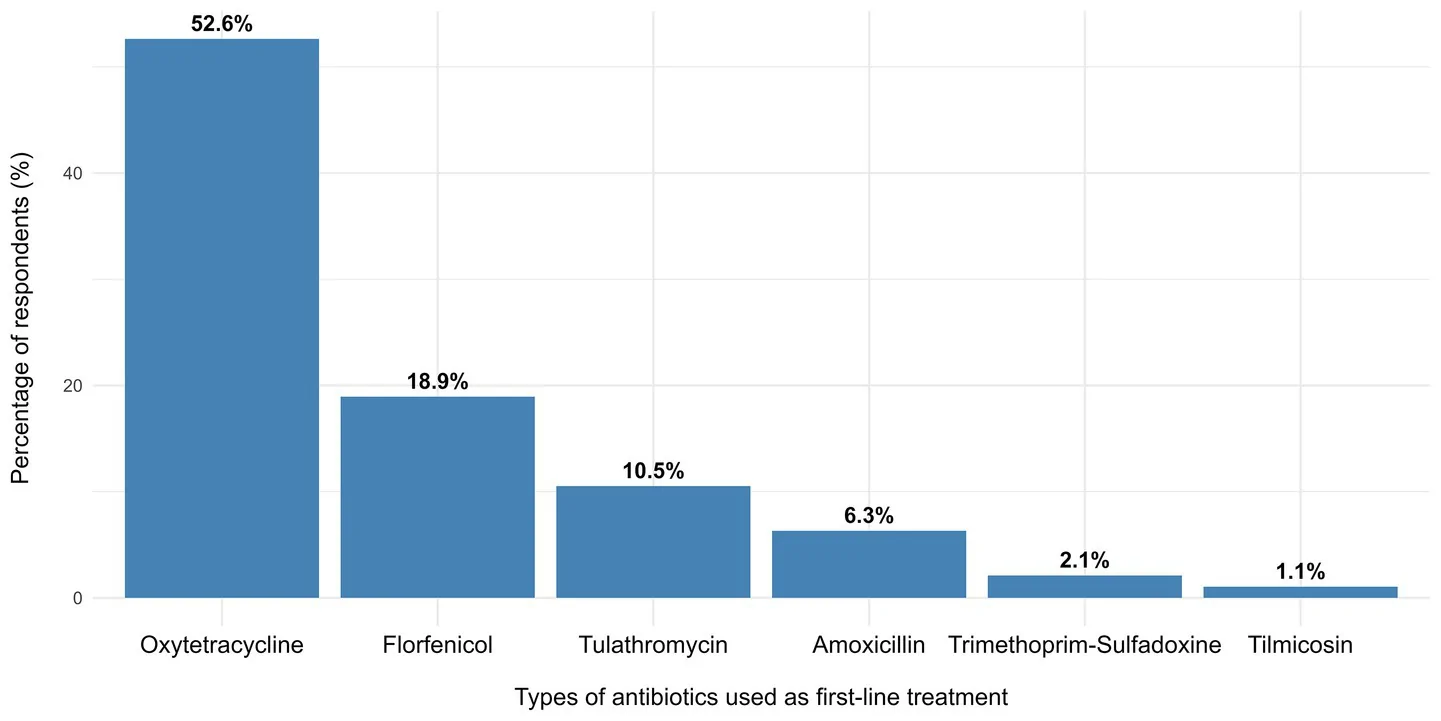
Proportion (%) of respondents selecting each antibiotic for first-line BRD therapy in calves (*n* = 95). Oxytetracycline was the most selected option (*n* = 50, 52.6%), followed by florfenicol (*n* = 18, 18.9%) and tulathromycin (*n* = 10, 10.5%).

**Figure 2:**
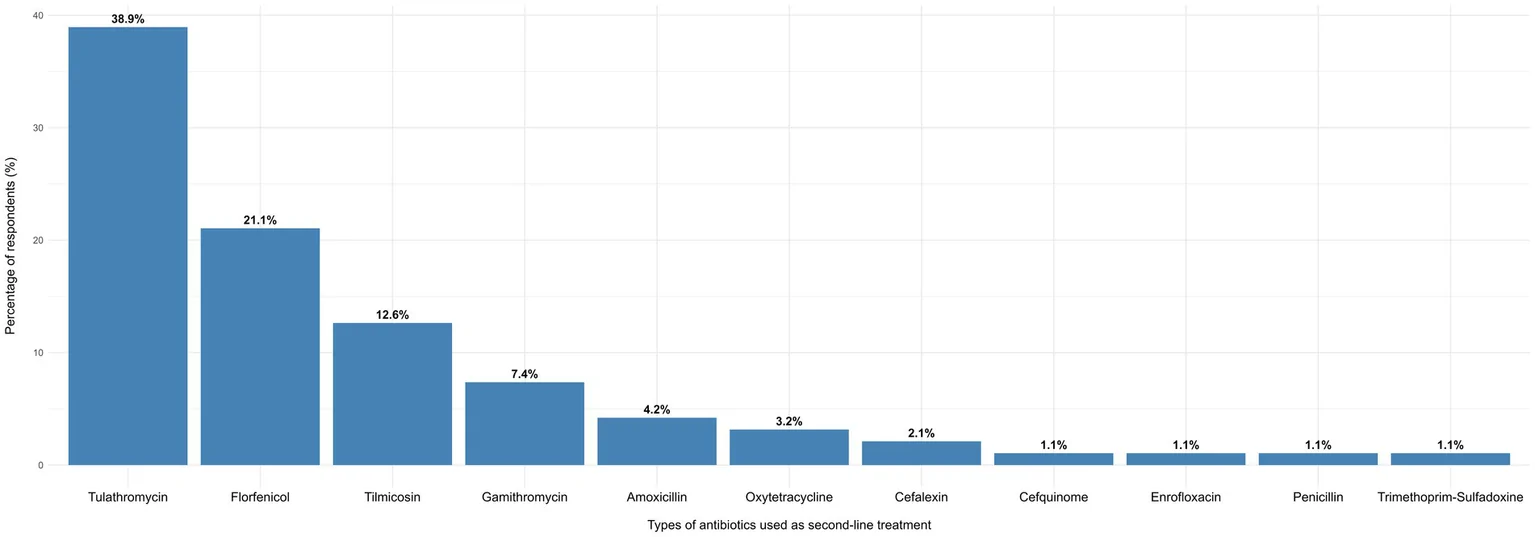
Percentage of respondents selecting each antibiotic as their preferred second-line treatment for BRD in calves (*n* = 95). Tulathromycin was the most frequently selected (*n* = 37, 38.9%), followed by florfenicol (*n* = 20, 21.1%) and tilmicosin (*n* = 12, 12.6%).

Most veterinarians (*n* = 57, 60.0%) administered a single course of antibiotic therapy for each BRD case until clinical signs improved, while approximately 42 (44.2%) waited 24 to 48 h to assess the effectiveness of the therapy. First-line antibiotic use, as shown in Figure 1, showed oxytetracycline (*n* = 50, 52.6%) as the most common initial choice. For second-line treatment, tulathromycin (*n* = 37, 38.9%) was most frequently selected, as depicted in Figure 2. The most common preventive measure did not require antibiotics (*n* = 47, 49.5%). A more detailed analysis revealed that, for first-line antibiotics, oxytetracycline (*n* = 70, 73.7%), florfenicol (*n* = 67, 70.5%), and tulathromycin (*n* = 53, 55.8%) were most frequently selected. For second-line therapy, tulathromycin (*n* = 66, 69.5%), florfenicol (*n* = 46, 48.4%), and tilmicosin (*n* = 31, 32.6%) were the main choices. Regarding preventive measures, non-antibiotic options were most often selected (*n* = 54, 56.8%), followed by oxytetracycline (*n* = 43, 45.3%) and tulathromycin (*n* = 20, 21.1%).

**Figure 3:**
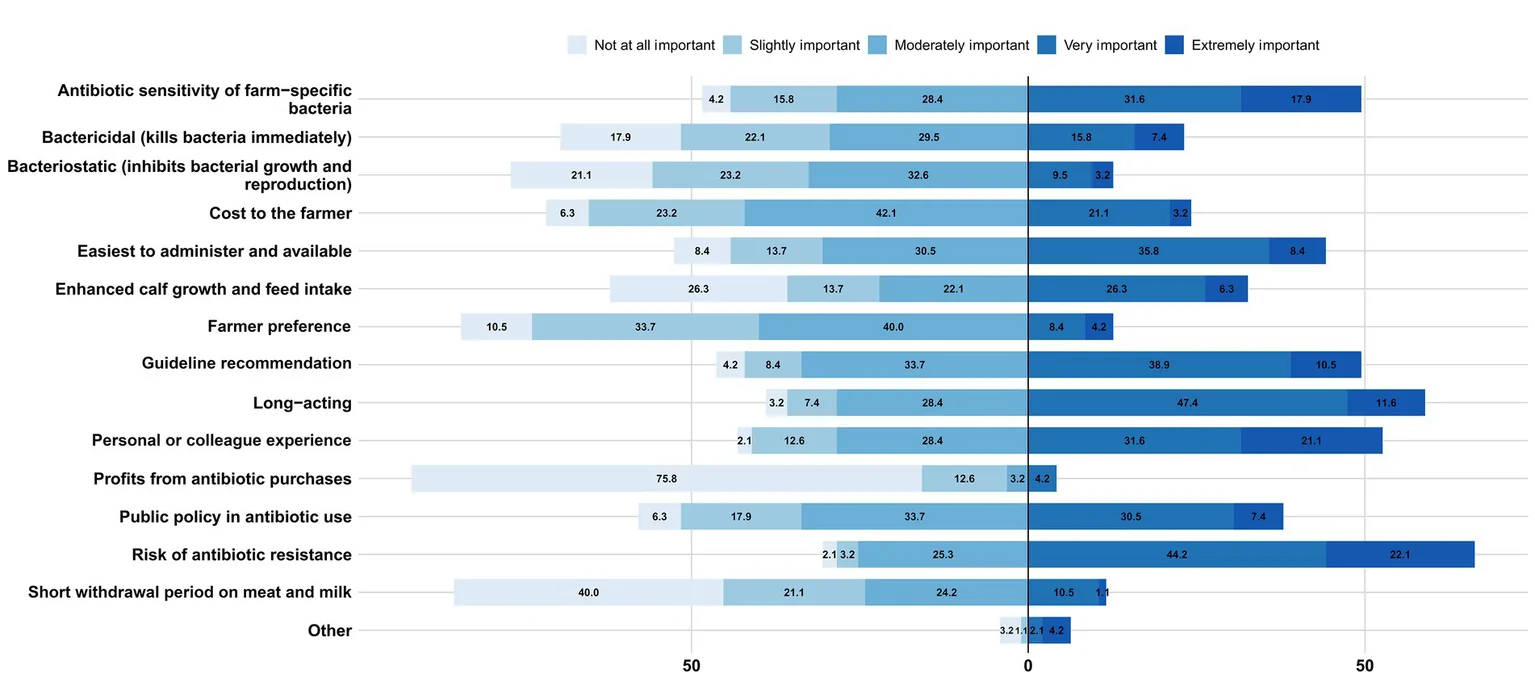
Horizontal bar chart showing the percentage of veterinarians rating each factor from “not at all important” to “extremely important” when selecting first-line antibiotics for BRD in calves (*n* = 95). Risk of antibiotic resistance was most frequently rated as “extremely important,” while profit from antibiotic purchases was most often rated as “not at all important”.

Based on Figure 3, the most important factors that influenced first-line antibiotic choice for BRD in calves were risk of antimicrobial resistance (AMR; *n* = 21, 22.1%) and clinical experience (*n* = 20, 21.1%), both rated as extremely important. This focus on prior case knowledge also appeared in qualitative responses, particularly in the topic of antibiotic susceptibility testing (AST) of farm-specific bacteria, where decisions relied more on farm-level case outcomes than *in vitro* results. In addition, 17 (17.9%) respondents rated antibiotic sensitivity as extremely important. Topics of practicality were also evident: long-acting formulations were rated extremely important by 11 (11.6%) respondents, while ease of administration was rated extremely important by 8 (8.4%) respondents.

Broader AMS considerations emerged across topics, as shown in Table 2, with representative quotations illustrating factors guiding antibiotic selection during qualitative analysis. For example, respondents describing a shift to category D antibiotics directly associated their selection with responsible use and AMR risk. When focusing on efficacy, respondents stressed the value of evidence-based treatment, antibiotics with proven effectiveness against BRD, and proper regulatory approval. In contrast, when clinical considerations or AMS were not prioritised, factors such as short withdrawal periods for meat and milk were consistently deemed unimportant. Specifically, 38 (40.0%) respondents rated short withdrawal periods as not at all important, while 72 (75.8%) rated profit from antibiotic purchases as not at all important.

**Table 2:** Summary of topics regarding the question “Any additional thoughts or comments on your first-line antibiotic treatment for BRD in calves” (*n* = 24 responses).

Topic	Definition	Quote
Antibiotic susceptibility testing (AST) of farm-specific bacteria	The laboratory procedures used to assess the responsiveness of bacterial strains found in a particular farm environment are to various antibiotics.	*“Testing and sensitive it is the one normally leading in my antibiotic treatment choice for BRD in calves.”* *“Antibiotic sensitivity usually decided by previous on farm response of cases rather than in vitro lab testing.”*
Category D “Prudence”	Should be used as first-line options whenever appropriate, but always with prudence, only when medically justified.	*“Primary reasons I choose oxytet: it is cat D and is effective vs. most likely pathogens (incl. M. bovis which I see quite often).”* *“Utilisation of Cat D antibiotics.”*
Risk of antibiotic resistance	The ability of bacteria to survive and proliferate despite the presence of antibiotics that would normally inhibit or kill them.	*“Very important to discover antibiotic resistance.”* *“We do have a responsibility to think about AMR.”*
Long acting	An antibiotic designed to exert its effect over a prolonged duration.	*”Long acting products can involve less handling or stress to ill animals.”* *“It is readily accessible and has a long-acting form.”*
Efficacy	The ability of an antibiotic to achieve its intended therapeutic effect.	*“Ideally we should be using drugs which have been shown to have efficacy in BRD through research and as such are on license.”* *“Published evidence base regarding efficacy is the most thing for me.”*

#### 3.2.2 Non-antibiotic therapies

**Figure 4:**
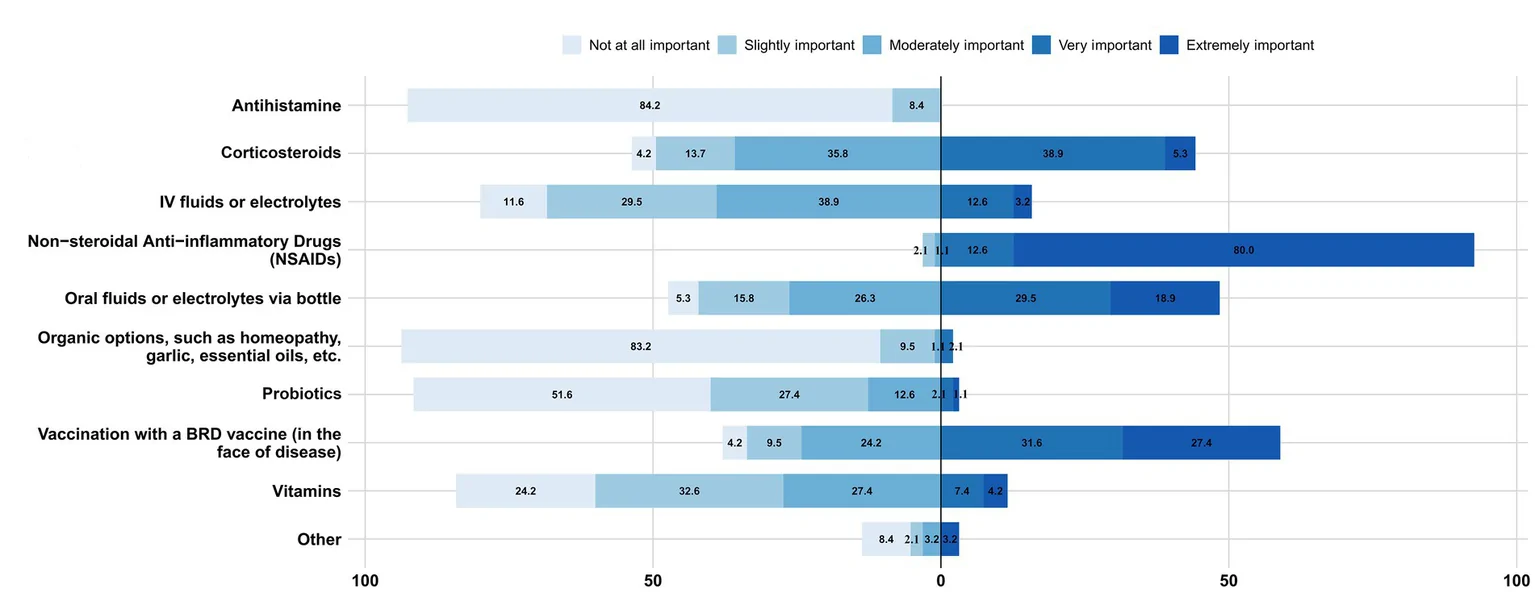
Responses reflected veterinarians’ evaluations of the importance of various ancillary treatments used alongside antibiotics for BRD in calves (*n* = 95). NSAIDs were most frequently rated as “extremely important,” while antihistamines were predominantly considered “not at all important”. IV, Intravenous.

Among respondents, 74 (77.9%) routinely combined non-antibiotic drugs with antibiotics as ancillary BRD therapies. In this context, Figure 4 showed that non-steroidal anti-inflammatory drugs (NSAIDs) are considered the most essential adjunct therapy, with 76 (80.0%) rating them as extremely important. Furthermore, qualitative responses reinforced this, as illustrated in Table 3, noting their use to reduce clinical signs, improve recovery, and promote calf welfare. Additionally, vaccination with a BRD vaccine (in the face of disease) was also highly rated: 30 (31.6%) rated it as very important and 26 (27.4%) as extremely important, particularly for timely benefits during outbreaks.

**Table 3:** Summary of topics regarding the question “Any additional thoughts or comments on the use of ancillary therapies for BRD in calves” (*n* = 23 responses).

Topic	Definition	Quote
Combination with non-steroidal anti-inflammatory drugs (NSAIDs)	Involves the concurrent use of antibiotics and anti-inflammatory agents to reduce inflammation, fever, and pain associated with infection, while eliminating bacterial pathogens.	*“I always use anti-inflammatory therapy in addition to antibiosis to treat BRD, though I tend go prescribe NSAIDs (due to faster onset of action & more favourable side effect profile).”* *“NSAIDs have been shown in the literature to improve recovery rates including welfare indicators such as how the calves behave and also a reduction in clinical signs, as well as decreasing longer term damage to the respiratory tract. They are the key aspect of BRD treatment that should be utilised in all incidences of BRD.”*
Fluid therapy	Fluids are administered intravenously or orally for hydration, electrolyte balance, and restoration or maintenance of circulatory function.	*“Supportive care (typically fluids) as needed depending on the clinical picture.”* *“I would recommend or administer fluid therapy in individual cases if it was indicated,* e.g.*, if there was concurrent scour and/or evidence of dehydration on clinical exam.”*
Vitamins	Organic compounds are required in small quantities for important metabolic functions, growth, and maintenance of health.	*“I have stated that vitamin status is because I have considerable clinical experience of neonatal vitamin A & E deficiencies, which are profoundly immunosuppressive, precipitating respiratory disease outbreak.”*
Vaccination with a BRD vaccine (in the face of disease)	The vaccines must be administered during an active outbreak or when animals are at risk of exposure, to reduce clinical severity, limit pathogen spread, and support recovery.	*“Only extremely when there is evidence the vaccine can help rapidly enough in acute outbreaks. Inactivated Mycoplasma bovis and M. haemolytica vaccines has also been effective anecdotally in creeping low symptom pneumonia.”*
Anthelmintic	An antiparasitic drug can be used to eliminate certain parasitic worms, including helminths.	*“In cases of verminous pneumonia, I advise anthelmintic treatment.”*

The topic of supportive care, specifically fluid therapy, emerged as beneficial, with oral fluids rated as very important (*n* = 28, 29.5%) and extremely important (*n* = 18, 18.9%). In qualitative responses, additional approaches, such as vitamins and anthelmintics, were described as contextual interventions, for example, used in herds with known deficiencies or in cases of verminous pneumonia. In contrast, some therapies were consistently rated as having limited importance. Antihistamines were rated as not at all important (*n* = 80, 84.2%), followed by organic treatments (*n* = 79, 83.2%) and probiotics (*n* = 49, 51.6%), and were generally considered to have only slight importance.

**Figure 5:**
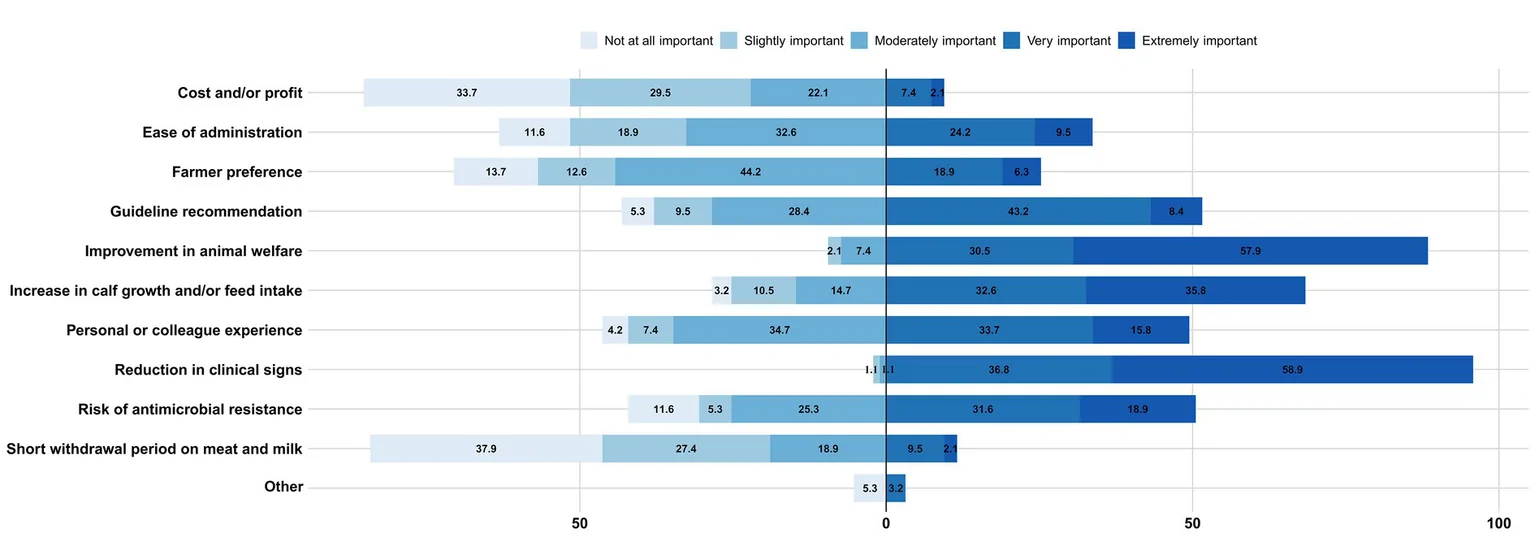
Importance ratings from veterinarians for reasons behind using non-antibiotic therapies to treat BRD in calves (*n* = 95). Reduction of clinical signs was most frequently rated as “extremely important,” while short withdrawal periods were most often rated as “not at all important”.

The most common reason for selecting ancillary therapies was the reduction of clinical signs, with 56 (58.9%) of respondents rating this as extremely important. Improved well-being (*n* = 55, 57.9%) and growth (*n* = 34, 35.8%) followed, as shown in Figure 5. Furthermore, AMR risk factors (*n* = 18, 18.9%), experience-based reasons (*n* = 15, 15.8%), and ease of administration (*n* = 9, 9.5%) were also identified as extremely important. By comparison, the most frequently rated as not at all important were brief withdrawal periods (*n* = 36, 37.9%) and cost considerations (*n* = 32, 33.7%).

#### 3.2.3 Control measures

Prevention and management of BRD in calves relied on several significant measures. Colostrum management received the highest rating for extreme importance (*n* = 76, 80.0%), with ventilation also extremely important (*n* = 62, 65.3%). Qualitative responses, as presented in Table 4, emphasised the value of dry housing and appropriate infrastructure. Group housing based on age groups was rated as extremely important (*n* = 34, 35.8%), particularly to minimise mixing between calves of different age groups. Vaccination programmes, both intranasal (*n* = 44, 46.3%) and injectable (*n* = 38, 40.0%), were considered extremely important, with vaccine selection influenced by age. In contrast, medicated water (*n* = 74, 77.9%), milk (*n* = 70, 73.7%), feed (*n* = 67, 70.5%), and group medication (*n* = 38, 40.0%) received low importance ratings. Additional comments highlighted broader prevention concerns, including the roles of nutrition, vitamin status, stress management, delayed disbudding, castration, and careful weaning.

**Table 4:** Summary of topics regarding the question “Any additional thoughts or comments on control measures for the prevention of BRD in calves” (*n* = 25 responses).

Topic	Definition	Quote
Vaccination programme for administering intranasal and injectable vaccines	A calculated timed administration for respiratory vaccines for cattle at risk for BRD through injectable or intranasal routes.	*“Vaccine use depends on age of the animal - intranasal often used in younger animals with injectable in older animals.”* *“I am highly in favour of respiratory disease vaccination however I find that the efficacy of this tends to vary considerably between farms.”*
Ventilation and facilities upgrades	Improving housing environments systematically, particularly airflow, air quality, and structural design, reduces airborne pathogen load, minimises stress-induced immunosuppression, and improves overall respiratory health in calves.	*“Ventilation, dry housing, extremely important.”* *“Adequate facilities needed - most farmers know when they are overcrowded and if the weather has contributed to making the housing insufficient, normally in November.”*
Group housing based on age groups	Calves are often housed in terms of cohorts of similar age.	*“Grouping of animals of similar ages in the same air space and avoiding mixing groups/shared airspaces with mature cattle are also key in controlling BRD.”* *“Also housing calves in groups of no more than 10 seems to be a key factor to success with infectious BRD.”*
Nutrition	Organisms obtain, assimilate, and utilise nutrients from food through biological processes to support growth, maintenance, reproduction, and overall health.	*“Nutrition & vitamin status can be of great importance.”* *“Good nutrition of the calves.”*
Stress management	Using physiological, environmental, and behavioural strategies lessens the detrimental effects of stress.	*“Stress management (*e.g.*, delayed disbudding, castration, weaning etc).”* *“Management of stressors - group changes, disbudding, weaning correctly.”*
Concurrent disease	In the same animal, two or more distinct pathological conditions are present simultaneously.	*“Coccidia, BVD causing immunosuppression.”* *“Check for BVD circulating.”*
Colostrum management	Colostrum of adequate quantity and quality is essential for calves at birth to support passive immunity, energy, and early health.	*“Always encourage excellent colostrum management.”*

### 3.3 Social and relational dynamics factors

Most respondents showed greater willingness to prescribe antibiotics when they believed farm staff used antibiotics appropriately (*n* = 65, 68.4%). The presence of a positive relationship with the farmer increased the likelihood of prescribing (*n* = 40, 42.1%). Meanwhile, a large proportion reported concern over potential blame from farmers as a motivating factor increasing the likelihood of prescribing (*n* = 43, 45.3%). Competitive pressure in the veterinary or farming community had a minimal impact and was neutral (*n* = 63, 66.3%). Besides, the majority of respondents (*n* = 40, 42.1%) frequently considered poor clinical response to antimicrobial administration when deciding on antibiotic use for BRD treatment, while some did so occasionally (*n* = 48, 50.5%). Antibiotic susceptibility testing (AST) was performed frequently by only a minority (*n* = 23, 24.2%), and the possibility that poor clinical response was due to AMR was often considered (*n* = 28, 29.5%).

## 4 Discussion

The literature describing UK farm veterinarians’ decision-making regarding antibiotic use for treating BRD in preweaned calves is limited. A previous UK study (2) focused on validating diagnostic methods and developing risk assessment tools for BRD in dairy herds, rather than on veterinarians’ treatment decisions. This study describes current diagnostic tools, explores veterinarians’ rationale for antibiotic and non-antibiotic choices, and identifies knowledge and gaps that impede effective management of BRD in dairy production. The findings from this study will help provide an evidence base for the development of future interventions to enhance AMS and mitigate AMR in UK dairy systems.

### 4.1 Demographic analysis

The demographic profile of respondents closely mirrored national patterns, despite showing subtle differences, and reinforcing the validity of the sample. The age distribution of respondents showed that the majority were in the 35–44 age group. This distribution aligns broadly with the Royal College of Veterinary Surgeons (RCVS) demographic data (36), which indicates that approximately half of all UK-practising veterinarians fall within the 25 to 44 age range, with the largest national cohorts being 26 to 30 and 31 to 35 years.

Regarding gender distribution, female respondents accounted for 58.9% of total respondents. This reflects the latest RCVS data (36), which shows a national workforce that is 66.8% female and 33.1% male in the UK. These figures reflect a wider shift across international veterinary medicine. For instance, female veterinarians now make up 65% of Europe’s workforce (37) and the similar US study (9) also reported a 51% female majority which highlights a changing profession where the current workforce is predominantly female (36).

Understanding the professional implications of this demographic shift is critical, particularly concerning future AMS strategies in agriculture. Sociological studies on UK farm veterinary communication (38) indicate that modern advisory roles heavily depend on establishing deep, trust-based partnerships and collaborative communication with farmers. A recent conceptual review by Batheja et al. (39) supports this shifting. Their work shows that gender norms and varying risk tolerances actively shape the economic and health choices surrounding antimicrobial use, and female practitioners tend to prescribe fewer antibiotics than male counterparts. Therefore, as female farm veterinarians often navigate clinical uncertainty in traditionally male-dominated agricultural settings, they may rely heavily on relationship-building to manage professional risks and farmer pressure. The strong influence of mutual trust and fear of blame on prescribing choices (Section 4.5) may explain this changing demographic. However, the overall demographic shift indicates our approach to AMS must evolve by recognising psycho-social factors. Interventions need to move beyond clinical guidelines and actively leverage relational approaches with the farmers.

While the sample size is modest relative to the total UK veterinary register, it is adequate for the exploratory objectives of this first UK study to specifically investigate antibiotic decision-making for BRD in preweaned calves. This approach aligns with similar research in the US (9) and the UK, where recent surveys of cattle veterinarians have successfully utilised sample sizes of 36 to 97 participants to capture focused professional perspectives (40, 41). Consequently, this sample is sufficient and maintains clinically meaningful associations, supporting robust descriptive statistics by representing 16.5% of the accessible, consented population. Ultimately, this study prioritises internal study validity and representativeness over achieving a large sample size that balances practical feasibility with methodological rigour (42).

### 4.2 Diagnostic approaches

Diagnosing BRD in calves is a crucial step in determining treatment course, selecting appropriate antibiotics, and developing prevention strategies. The results of this study demonstrate a decisive preference for pragmatic, field-friendly diagnostic practices over advanced technologies. By prioritising rectal temperature and clinical examination, veterinarians are selecting tools based on speed, cost-effectiveness, and ease of use in rural settings (43). This reliance on low-tech methods aligns with Chan et al. (10), suggesting that while veterinarians recognise the value of laboratory data, the reality of field conditions, specifically budget constraints and the need for immediate decision-making, prevents the adoption of more sophisticated methods.

This observation differs from Mijares et al. (9), who ranked on-farm necropsy as the most “extremely important” diagnostic tool. This may represent cultural, structural, and diagnostic differences between US and UK veterinary farm practice. Reliance on post-mortem confirmation can delay early treatment after clinical signs are observed. Instead, the rectal temperature preference in this study may better reflect a more proactive attempt at early detection (44, 45). However, it must be noted that relying solely on fever is limited, suggesting that both low-tech approaches may still fail to accurately diagnose BRD incidence within a dairy herd.

In the context of AMR, this diagnostic limitation is a significant concern. Decisions based solely on clinical observation or fever risk increase inappropriate antibiotic use (25, 46). As suggested by Kamel et al. (3), veterinarians should be encouraged to integrate field-relevant instruments with contextual farm history and seasonal factors to enhance diagnostic confidence. Consequently, future work should seek to improve the availability of bedside tests, such as rapid digital thermometers, lung ultrasound, and respiratory scoring charts, to bridge the gap between field practicality and diagnostic accuracy.

Interestingly, our findings did not reveal a demographic divide in the adoption of advanced diagnostic technologies. While one might expect younger veterinarians to naturally prefer tools like ultrasound, our data showed no significant correlation. It appears that on-the-ground practical limitations simply override generational differences. Recent literature focusing on UK dairy farms highlights that antemortem diagnosis of BRD remains complex, and the diagnostic accuracy of routine clinical respiratory scoring is still imperfect when compared directly to Thoracic Ultrasonography (TUS) on-farm (2). Therefore, the continued reliance on rapid, standard methods seems to be driven more by logistical realities and disease complexity rather than a practitioner’s age or graduation year.

Regarding standard clinical examinations, our initial observation of demographic variations highlighted the nuanced nature of field diagnostics. However, our targeted binary analyses revealed a striking consensus: neither binary gender (male vs. female) nor specific experience cohorts (<6 years vs. ≥6 years) significantly altered the high value placed on these fundamental hands-on methods. While a recent nationwide survey (47) demonstrated that human factors, specifically gender and clinical experience, significantly shape the perception of disease severity and intervention thresholds, our data suggests this divergence does not extend to the valuation of foundational diagnostic tools. Instead, the universal reliance on clinical examination across the main demographic groups suggests that the practical demands of bovine practice override these individual differences. Furthermore, although recent findings (48) indicate that accumulated experience shapes broader management strategies, our results show that both junior and senior practitioners equally lean on structured, hands-on data collection to reduce diagnostic uncertainty in unpredictable farm environments.

### 4.3 Patterns of antibiotic selection for BRD treatment

Oxytetracycline was the predominant first-line antibiotic therapy. This reliance on tetracyclines, phenicols, and macrolides appears driven by their broad-spectrum activity, established clinical efficacy, ease of administration, and affordability (25, 49). These results align with Mijares et al. (9), who reported that clinical experience is the primary factor in determining antibiotic choice, and with a preference for long-acting formulations that minimise animal handling (4).

When treating BRD cases that did not resolve with first-line therapy (usually assessed between 24 and 48 h), respondents reported switching to a different antibiotic class in accordance with infection control recommendations to prevent AMR (24, 26). Tulathromycin is reported to be the most frequently used second-line choice due to its high lung tissue penetration and efficacy in severe or relapsing cases (50). Mijares et al. (9) reported that antibiotic switching is common in BRD cases, yet this practice contributes significantly to overall antibiotic use. While our respondents’ use of a potent macrolide like tulathromycin is clinically logical, it highlights the challenge raised by Mijares et al. (9) that veterinarians must balance immediate clinical efficacy with responsible use, particularly given that retreatment significantly increases the total antibiotic load.

Regarding prevention, the results reveal a dichotomy in practice. Nearly half of respondents did not use prophylactic antibiotics, signalling a positive shift towards AMS and non-antibiotic prevention strategies, such as vaccination and biosecurity. Conversely, among those practising metaphylaxis, oxytetracycline remained the most frequent choice. This continued reliance on antimicrobials contradicts current global recommendations to restrain antimicrobial use (24, 51, 52). While some studies suggest long-acting macrolides reduce BRD incidence in high-risk calves (53), the routine use of oxytetracycline suggests that economic constraints and perceived safety profiles (25, 54) still frequently outweigh AMS concerns in field settings. This reflects the tension observed in calf-rearing operations (55), where reliance on the product often persists despite evidence supporting alternative preventive strategies.

### 4.4 Veterinarians’ rationale for antibiotic choices and alternative treatments

The primary factors influencing the choice of first-line BRD antibiotics in preweaned UK calves are AMS and clinical experience. In this study, 22.1% of respondents prioritised the risk of AMR, and 21.1% prioritised clinical experience, both ranking above commercial concerns such as profit or withdrawal periods. These findings align with the professional culture described by Powell et al. (56), which found that veterinarians favour profession-led stewardship, such as internal training and clinical clubs, rather than external regulation or punitive measures. The results suggest that the profession is characterised by clinical autonomy and stewardship, in accordance with international AMS guidance on antibiotic use. On the other hand, because our sample heavily features UK farm veterinarians already engaged in AMS networks, these data likely reflect a best-case scenario (as discussed in Section 4.6). Consequently, they may not represent the baseline prescribing habits of the wider veterinary community. However, a tension exists between empirical experience and evidence-based protocols. While respondents prioritised bacterial susceptibility, many still relied on empirical selection, choosing high-efficacy, long-acting macrolides like tulathromycin or tildipirosin to reduce treatment frequency and animal stress, consistent with previous studies (57, 58). This mirrors the findings of Monteiro et al. (59), who found that practitioners favoured empirical success over waiting for AST results. Although Feßler et al. (60) argued that AST produces equivalent clinical outcomes with lower resistance risks, the results of the current study suggest that the transition from empirical to AST-led therapy in the UK is hindered more by a lack of rapid diagnostic infrastructure rather than by professional reluctance.

Our study findings demonstrate that 40% of the respondents considered meat and milk withdrawal times in their antibiotic choices as “not at all important”. Because our study focuses specifically on preweaned calves, this variable was included primarily to mirror standardised prescribing factors used in broader UK dairy studies (30). The resulting low score logically reflects the clinical reality of these young animals that they are non-lactating and are a long way from slaughter, meaning they pose no immediate risk to the food supply chain. While veterinarians across the UK typically must pay close attention to withdrawal periods when treating adult cattle (30), clinical decisions for preweaned calves are driven by therapeutic efficacy and past clinical experience (9). Therefore, veterinarians have the flexibility to focus on AMS and animal welfare rather than considering strict withdrawal timelines.

The rationale behind these prescribing choices in the UK also provides a notable contrast to other European systems, such as in Denmark. Recent research by Skjølstrup et al. (61) highlights that Danish veterinarians operate within a highly regulated environment, specifically the ‘Yellow Card’ scheme, which mandates the use of narrow-spectrum antimicrobials and limits clinical manoeuvring. In contrast, UK veterinarians maintain significant clinical autonomy, allowing for a higher reliance on broad-spectrum options like oxytetracycline and tulathromycin based on perceived clinical efficacy rather than legislative pressure. However, despite these different regulatory landscapes, similarities exist in the social drivers of prescribing. Much like our UK cohort, Danish practitioners navigate complex, negotiated relationships with farmers and often perceive AMR as an abstract, global threat rather than a local clinical reality. This suggests that while national policy can dictate the choice of molecule, the underlying behavioural barriers and relational dynamics between veterinarians and farmers are a consistent international challenge to AMS.

A critical finding of this study is the high value placed on non-antibiotic therapies, specifically NSAIDs, which were considered “extremely important” by 80% of respondents. In contrast, a prior study by Mijares et al. (9) found that only 30% of respondents rated NSAIDs as “extremely important,” and noted that cost, extra-label use, and farmer resistance were obstacles, even though they recognised the pain related to BRD. Building on this, the results of this study suggest a more established multimodal professional standard in the UK. The support for NSAIDs here aligns with recent studies (4, 62), which noted that suppressing the inflammatory cascade is essential for limiting subsequent pulmonary lesions and improving welfare.

Despite this high perceived importance, the actual administration of supportive care remains conditional. Consistent with the study findings of Mijares et al. (9), UK respondents indicated that analgesia use is often dependent on the perceived severity of the case and the farmer’s willingness to administer additional treatments. This suggests a persistent knowledge-action gap; while UK veterinarians recognise the welfare necessity of pain relief more acutely than the cohorts in the Mijares et al. (9) study, practical application remains influenced by farm-level logistics.

Nevertheless, the reported use of supportive therapies, such as oral fluids and vitamins, reflects a clear understanding of BRD as a multifactorial disease. This aligns with literature emphasising that environmental and nutritional management are as vital as pharmacological interventions (3, 62, 63). Clinical evidence shows that calves receiving oral hydration alongside standard care experience higher cure rates and fewer chronic infections (64). Furthermore, maintaining the structural integrity of respiratory cells and driving a robust immune response requires specific nutritional support, particularly vitamins A and E (65, 66). Prioritising these non-antibiotic interventions to accelerate clinical recovery provides practitioners with a highly practical mechanism to scale back overall antimicrobial usage for mitigating AMR.

Moreover, other findings from this current analysis support an integrated preventive strategy, including colostrum management and ventilation upgrades as the most important non-pharmaceutical interventions. This was consistent with some other studies (63, 67), confirming that the main BRD morbidity defence strategies were optimising passive immunity and air quality. It also suggests that vaccination is considered a prevention culture to reduce reliance on routine antimicrobial usage (26). While these findings mirror the integrated biosecurity models proposed by Mijares et al. (9), the current study cites initial costs and a lack of facilities as common barriers to proper implementation (68). Other social management practices, such as calf segregation, stocking density, and nutritional support, can reduce the effects of stress and disease transmission. These observations support the findings observed in earlier papers by Lemon and McMenamy (69) and Richeson (70), who argued that management-based prevention is the most effective tool for reducing antibiotic reliance.

To bridge the gap between empirical treatment and sustainable AMS, future approaches should prioritise integrating rapid pen-side diagnostics into BRD management. Nickell et al. (71) demonstrated that adopting risk-based targeted therapy can reduce broad-spectrum antibiotic use up to 43% while maintaining animal health outcomes. In the future, the UK veterinary profession may consider combinations of antibiotics and responsible NSAID use to optimise clinical outcomes and animal welfare, and to reduce the industry’s dependence on Highest Priority Critically Important Antimicrobials (HP-CIAs) (25, 51).

### 4.5 Social and relational dynamics factors

Our results show that BRD prescribing decisions rely on veterinarian-farmer trust and not solely on clinical factors. Veterinarians frequently prescribed because they believed farm personnel administered treatment properly, illustrating delegated authority, with social pressure and trust replacing direct clinical oversight. This agrees with Coyne et al. (25), who found the veterinarian-farmer relationship is a key reason for prescribing, and with Rees et al. (72), who noted that treatment decisions often result from negotiation between clinical advice and farmers’ experience. Our findings also reveal that the fear of farmer blame increased the likelihood of prescribing. This highlights the complexity of the relationship: a strong bond can facilitate prescribing, as veterinarians may still prioritise relationships over stewardship if they fear harming those ties. While Higgins et al. (73) suggested social factors are absent in high-regulation settings, our findings show that, in the absence of mandatory testing, relational trust often substitutes for formal diagnosis.

The discrepancy between the frequent observation of a poor clinical response and the rare use of AST in clinical practice appears to result from two factors. First, most respondents did not consider AMR as a likely cause for treatment failure, reflecting a disconnect between theoretical awareness of AMR and its perceived local impact, which acts as a significant barrier. While practitioners theoretically accept AMR as a global threat, they rarely attribute failure in their own caseloads to resistance, viewing it instead as an issue of *ad hoc* management (74, 75). Second, ingrained habits and clinical urgency frequently take precedence over diagnostics, particularly when laboratories are slow to process, as aligns with Coyne et al. (25).

These barriers are primarily behavioural (habitual non-use of AST) and social (fear of blame and reliance on trust), which implies that traditional education alone is insufficient. Future effective interventions within the One Health framework must consider these critical behavioural and social factors, for example, using frameworks like the Behaviour Change Wheel (BCW) (76) and Self-Determination Theory (77). Using the BCW, specific intervention tools such as training can be implemented to enhance both the veterinarians’ capability to integrate AST into daily routines, and their psychological capability for effective communication. In parallel, peer modelling and environmental restructuring can address motivational barriers and social constraints, such as the fear of farmer blame. Our results identify two priorities for future research: (a) Communication training to help veterinarians preserve farmer trust and manage fear of blame without relying on antimicrobial prescriptions as the professional influence; and (b) Evaluating behavioural changes for financial incentives or regulations that mandate AST optimisation before changing antimicrobial classes. Therefore, focusing on these areas could better align veterinarians’ practice with AMS goals.

### 4.6 Study limitations

Selection bias from non-probabilistic sampling is a notable limitation in our study, as our respondents might not perfectly represent all UK dairy veterinarians. Specifically, just over half of the cohort (52.6%) were affiliated with the Veterinary Prescribing Champions Network. It is highly likely that those who volunteered, particularly those active in such networks are already heavily invested in calf health and AMS. These findings might have created a best-case scenario for prescribing practices, reflecting the views of a more engaged subpopulation rather than the true baseline of the entire UK farm-veterinarian population. This has some implications for relating to the generalisability of these results (34, 42). Additionally, we recognise that a single cross-sectional survey cannot examine the complexities of on-farm clinical decision-making. To explore these nuances more deeply, subsequent qualitative investigations, such as focus groups or interviews are highly recommended.

Furthermore, because this survey was explicitly designed to evaluate the behavioural and perceptual drivers of prescribing, focusing on why UK farm veterinarians make certain decisions, so we did not collect quantitative statistics or clinical efficacy data comparing non-antibiotic adjunctive therapies (e.g., NSAIDs, fluid therapy, vitamins) against antibiotic use. While understanding the clinical effectiveness of these non-antibiotic strategies is critical for formulating robust AMR mitigation strategies, evaluating comparative clinical outcomes fell outside our methodological scope. This highlights a critical area for subsequent studies to address. These descriptive results should therefore be interpreted with appropriate caution, keeping the boundaries of the study design in mind.

## 5 Conclusion

In conclusion, this study offers novel insights into the diagnostic approaches, treatment practices, and social factors influencing UK farm veterinarians’ management of BRD in preweaned calves. The results show a predominant reliance on clinical signs for diagnosis, frequent use of oxytetracycline and NSAIDs as first-line antimicrobials, and the most valued ancillary therapy. The selection of antibiotics was influenced by several factors, including concern regarding AMR, professional experience, and adherence to guidelines. Preventive measures, including colostrum management, ventilation, and vaccination, were commonly implemented, although laboratory confirmation through bacterial culture and sensitivity testing remained limited. Social pressures, particularly trust in farm staff and fear of farmer blame, also played a significant role in this process. The results outline the intricate interplay among clinical expertise, social dynamics, and policy frameworks in antimicrobial prescribing. The enhancement of diagnostic capacity, the inclusion of evidence-based preventive strategies, and improving veterinarian-farmer communication through interventions based on behavioural models like the Behaviour Change Wheel, for example training, modelling, and environmental restructuring, were identified as crucial elements in elevating AMS and the health of calves. Future research should explore the potential for targeted interventions to address both technical and behavioural drivers of antibiotic use. These interventions could support more sustainable livestock health management in the UK dairy sector.

## Data Availability

The raw data supporting the conclusions of this article will be made available by the authors, without undue reservation.
