# Correction: Understanding antibiotic decision-making for bovine respiratory disease: a survey of UK farm veterinarians

**DOI:** 10.3389/fvets.2026.1900336

**Published:** 2026-07-16

**Authors:** Ardi B. Prakoso, Gwenllian M. Rees

**Affiliations:** 1 Department of Life Sciences, Aberystwyth University, Aberystwyth, Ceredigion, United Kingdom; 2 Department of Life Sciences, School of Veterinary Science, Aberystwyth University, Aberystwyth, Ceredigion, United Kingdom

**Keywords:** antibiotic prescribing behaviour, antimicrobial stewardship, bovine respiratory disease, diagnostic and preventive practices, veterinarian-farmer relationships

The standard abbreviation for the country name upon first mention was omitted. A correction has been made to the section **Introduction**, Paragraph 6.

The sentence “In the United States, cross-sectional surveys of veterinarians managing BRD in preweaned dairy calves showed widespread use of antibiotics as first-line treatment, most commonly tulathromycin, with florfenicol as a frequent second-line choice.” has been corrected to “In the United States (US), cross-sectional surveys of veterinarians managing BRD in preweaned dairy calves showed widespread use of antibiotics as first-line treatment, most commonly tulathromycin, with florfenicol as a frequent second-line choice.”

The standard abbreviation for the country name was added to another instance to maintain consistency. A correction has been made to the section **Materials and methods**, *subsection 2.1*, Paragraph 1:

The sentence “The survey design was based on research by Mijares et al. (9), which investigated farm veterinarians' perspectives on treatment and diagnostic tools related to BRD in preweaned calves in the United States.” has been corrected to “The survey design was based on research by Mijares et al. (9), which investigated farm veterinarians' perspectives on treatment and diagnostic tools related to BRD in preweaned calves in the US.”

There was a typographical error in the respondent count, a minor rounding decimal, an inconsistency in the italicisation of Latin terms, and a need to separate figure citations accurately.

A correction has been made to the section **Results**, Paragraph 1. The sentence “Geographically, most were based in England (*n* = 43, 45.3%) and Wales (*n* = 40, 42.1%).” has been corrected to “Geographically, most were based in England (*n* = 44, 46.3%) and Wales (*n* = 41, 43.2%).”

A correction has been made to the section **Results**, *subsection 3.1*, Paragraph 5:

The sentence “Nevertheless, when specifically comparing veterinarians with less than 6 years of experience to those with six or more years, the post-hoc OR indicated no statistically significant difference between these specific cohorts (OR = 0.835; *p* = 0.725).” has been corrected to “Nevertheless, when specifically comparing veterinarians with less than 6 years of experience to those with six or more years, the *post-hoc* OR indicated no statistically significant difference between these specific cohorts (OR = 0.835; *p* = 0.725).”

A correction has been made to the section **Results**, *subsection 3.2.1*, Paragraph 1. The sentence “Most veterinarians (*n* = 57, 60.0%) administered a single course of antibiotic therapy for each BRD case until clinical signs improved, while approximately 39 (44.2%) waited 24 to 48 h to assess the effectiveness of the therapy.” has been corrected to “Most veterinarians (*n* = 57, 60.0%) administered a single course of antibiotic therapy for each BRD case until clinical signs improved, while approximately 42 (44.2%) waited 24 to 48 h to assess the effectiveness of the therapy.”

A correction has been made to the section **Results**, *subsection 3.2.1*, Paragraph 1. The sentence “First-line antibiotic use, as shown in Figures 1, 2, showed oxytetracycline (*n* = 50, 52.6%) as the most common initial choice. For second-line treatment, tulathromycin (*n* = 37, 38.9%) was most frequently selected.” has been corrected to “First-line antibiotic use, as shown in Figure 1, showed oxytetracycline (*n* = 50, 52.6%) as the most common initial choice. For second-line treatment, tulathromycin (*n* = 37, 38.9%) was most frequently selected, as depicted in Figure 2.”

A correction has been made to the section **Results**, *subsection 3.2.1*, Paragraph 1.

The sentence “For second-line therapy, tulathromycin (*n* = 66, 69.5%), florfenicol (*n* = 46, 48.4%), and tilmicosin (*n* = 31, 32.6%) were the main choices. Regarding preventive measures, non-antibiotic options were most often selected (*n* = 54, 56.8%), followed by oxytetracycline (*n* = 43, 45.3%) and tulathromycin (*n* = 20, 21.0%).” has been corrected to “For second-line therapy, tulathromycin (*n* = 66, 69.5%), florfenicol (*n* = 46, 48.4%), and tilmicosin (*n* = 31, 32.6%) were the main choices. Regarding preventive measures, non-antibiotic options were most often selected (*n* = 54, 56.8%), followed by oxytetracycline (*n* = 43, 45.3%) and tulathromycin (*n* = 20, 21.1%).”

There was an error in describing the data points accurately. A correction has been made to the section **Results**, *subsection 3.3*, Paragraph 1. The sentence “The presence of a positive relationship with the farmer reduced the likelihood of prescribing (*n* = 40, 42.1%). Meanwhile, the majority reported concern over potential blame from farmers as a motivating factor increasing the likelihood of prescribing (*n* = 43, 45.3%).” has been corrected to “The presence of a positive relationship with the farmer increased the likelihood of prescribing (*n* = 40, 42.1%). Meanwhile, a large proportion reported concern over potential blame from farmers as a motivating factor increasing the likelihood of prescribing (*n* = 43, 45.3%).”

Minor grammatical and typographical adjustments were implemented for clarity.

A correction has been made to the section **Discussion**, Paragraph 1:

Sentence “The findings from this study will help provide an evidence-base for the development of future interventions to enhance AMS and mitigate AMR in UK dairy systems.” has been corrected to “The findings from this study will help provide an evidence base for the development of future interventions to enhance AMS and mitigate AMR in UK dairy systems.”

A correction has been made to the section **Discussion**, *subsection 4.1*, Paragraph 3:

The sentence “A recent conceptual review by Batheja et al. (39) support this shifting.” has been corrected to

“A recent conceptual review by Batheja et al. (39) supports this shifting.”

A correction has been made to the section **Discussion**, *subsection 4.1*, Paragraph 4:

The sentence “Ultimately, this study priorities internal study validity and representativeness over achieving a large sample size that balances practical feasibility with methodological rigour (42).” has been corrected to “Ultimately, this study prioritises internal study validity and representativeness over achieving a large sample size that balances practical feasibility with methodological rigour (42).”

A correction has been made to the section **Discussion**, *subsection 4.4*, Paragraph 2. The sentence “Our study findings demonstrate that 40% of the respondents considered meat and milk withdrawal times into their antibiotic choices as “not at all important”.” has been corrected to “Our study findings demonstrate that 40% of the respondents considered meat and milk withdrawal times in their antibiotic choices as “not at all important”.”

A correction has been made to the section **Discussion**, *subsection 4.4*, Paragraph 4. The sentence “The support for NSAIDs here aligns with recent studies (4, 62), who noted that suppress the inflammatory cascade is essential for limiting subsequent pulmonary lesions and improving welfare.” has been corrected to, “The support for NSAIDs here aligns with recent studies (4, 62), which noted that suppressing the inflammatory cascade is essential for limiting subsequent pulmonary lesions and improving welfare.”

A correction has been made to the section **Discussion**, *subsection 4.4*, Paragraph 5. The sentence “This suggests a persistent knowledge-action gap, while UK veterinarians recognise the welfare necessity of pain relief more acutely than the cohorts in the Mijares et al. (9) study, practical application remains influenced by farm-level logistics.” has been corrected to “This suggests a persistent knowledge-action gap; while UK veterinarians recognise the welfare necessity of pain relief more acutely than the cohorts in the Mijares et al. (9) study, practical application remains influenced by farm-level logistics.”

A correction has been made to the section **Discussion**, *subsection 4.4*, Paragraph 6.

The sentence “Also, maintaining the structural integrity of respiratory cells and driving a robust immune response also requires specific nutritional support, particularly vitamins A and E (65, 66).” has been corrected to “Furthermore, maintaining the structural integrity of respiratory cells and driving a robust immune response requires specific nutritional support, particularly vitamins A and E (65, 66).”

There were a sentence fragment error and a need to align with corrected data in the **Results** section. A correction has been made to the section **Discussion**, *subsection 4.5*, Paragraph 1. The sentence “Our results show that BRD prescribing decisions rely on veterinarian-farmer trust and not solely on clinical factors. Veterinarians frequently prescribed because they believed farm personnel administered treatment properly, illustrating how delegated authority, with social pressure and trust replacing direct clinical oversight.” has been corrected to “Our results show that BRD prescribing decisions rely on veterinarian-farmer trust and not solely on clinical factors. Veterinarians frequently prescribed because they believed farm personnel administered treatment properly, illustrating delegated authority, with social pressure and trust replacing direct clinical oversight.”

A correction has been made to the section **Discussion**, *subsection 4.5*, Paragraph 1. The sentence “This highlights the complexity of the relationship: while a strong bond can lower prescribing, veterinarians may still prioritise relationships over stewardship if they fear harming those ties.” has been corrected to “This highlights the complexity of the relationship: a strong bond can facilitate prescribing, as veterinarians may still prioritise relationships over stewardship if they fear harming those ties.”

The original version of this article has been updated.

