# Correction: Understanding antibiotic decision-making for bovine respiratory disease: a survey of UK farm veterinarians

**DOI:** 10.3389/fvets.2026.1973864

**Published:** 2026-09-18

**Authors:** Frontiers Production Office

**Affiliations:** Frontiers Media SA, Lausanne, Switzerland

**Keywords:** antibiotic prescribing behaviour, antimicrobial stewardship, bovine respiratory disease, diagnostic and preventive practices, veterinarian-farmer relationships

There was a mistake in the captions of Tables 1–4, as published. The phrase “and percentage of responses” was erroneously included, as the tables only display topics, definitions, and quotes and do not contain percentage values. The corrected captions of Tables 1–4 appears below.

“Table 1

Summary of topics regarding the question “Please explain your answer further regarding the importance of these diagnostic tools of BRD in calves” (*n* = 44 responses).”

“Table 2

Summary of topics regarding the question “Any additional thoughts or comments on your first-line antibiotic treatment for BRD in calves” (*n* = 24 responses).”

“Table 3

Summary of topics regarding the question “Any additional thoughts or comments on the use of ancillary therapies for BRD in calves” (*n* = 23 responses).”

“Table 4

Summary of topics regarding the question “Any additional thoughts or comments on control measures for the prevention of BRD in calves” (*n* = 25 responses).”

File data sheet 1 was erroneously published with the original version of this paper. The file has now been replaced.

There was a mistake in Figure 2 as published. There was a typographical error on the x-axis where the drug name “Cefquinome” was misspelled as “Cequinome”. The corrected Figure 2 appears below.

**Figure 2:**
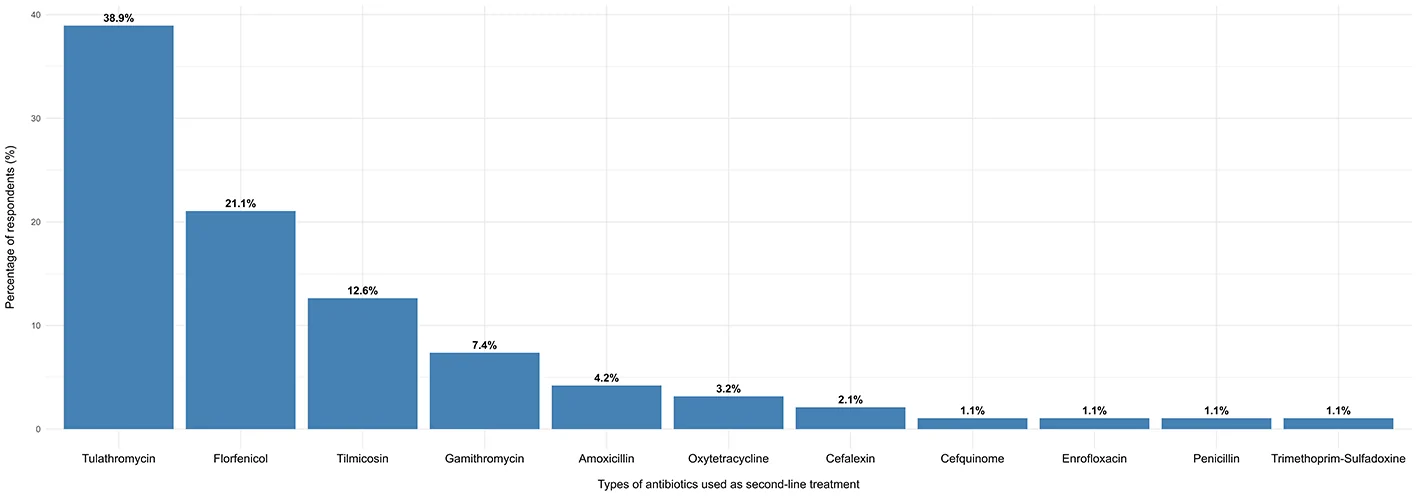
Percentage of respondents selecting each antibiotic as their preferred second-line treatment for BRD in calves (*n* = 95). Tulathromycin was the most frequently selected (*n* = 37, 38.9%), followed by florfenicol (*n* = 20, 21.1%) and tilmicosin (*n* = 12, 12.6%).

The original version of this article has been updated.

## Generative AI statement

Any alternative text (alt text) provided alongside figures in this article has been generated by Frontiers with the support of artificial intelligence and reasonable efforts have been made to ensure accuracy, including review by the authors wherever possible. If you identify any issues, please contact us.

