# Supplementary material for: Understanding antibiotic decision-making for bovine respiratory disease: a survey of UK farm veterinarians
Source: Front Vet Sci. 2026 May 19;13:1791479. doi: 10.3389/fvets.2026.1791479 (PMC13225968; doi:10.3389/fvets.2026.1791479)
Supplement: Supplementary file 1 [file Data_Sheet_1.docx]

Supplementary Material

# Supplementary Data

Further additional information and the full dataset of this research are provided in the Appendices.

# Appendices

#
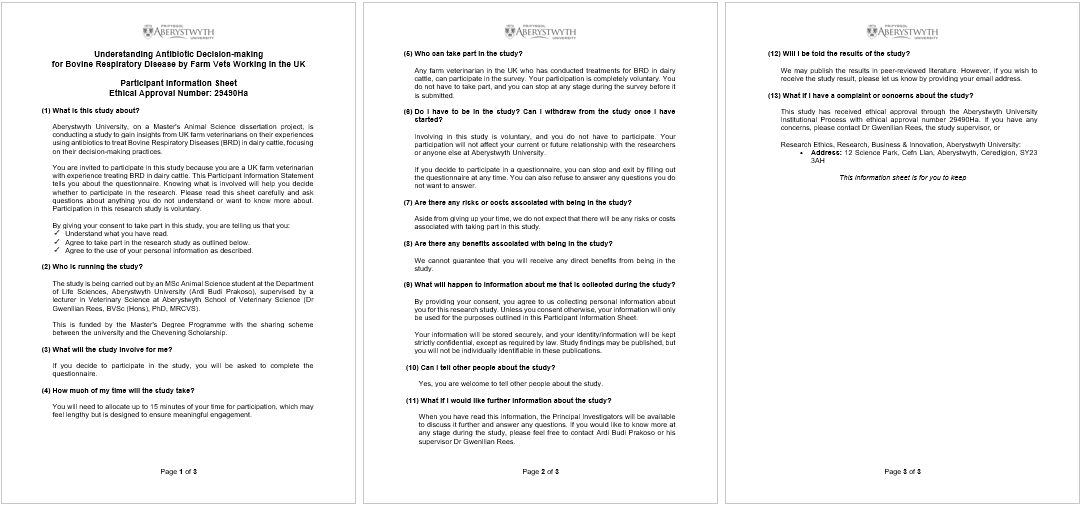


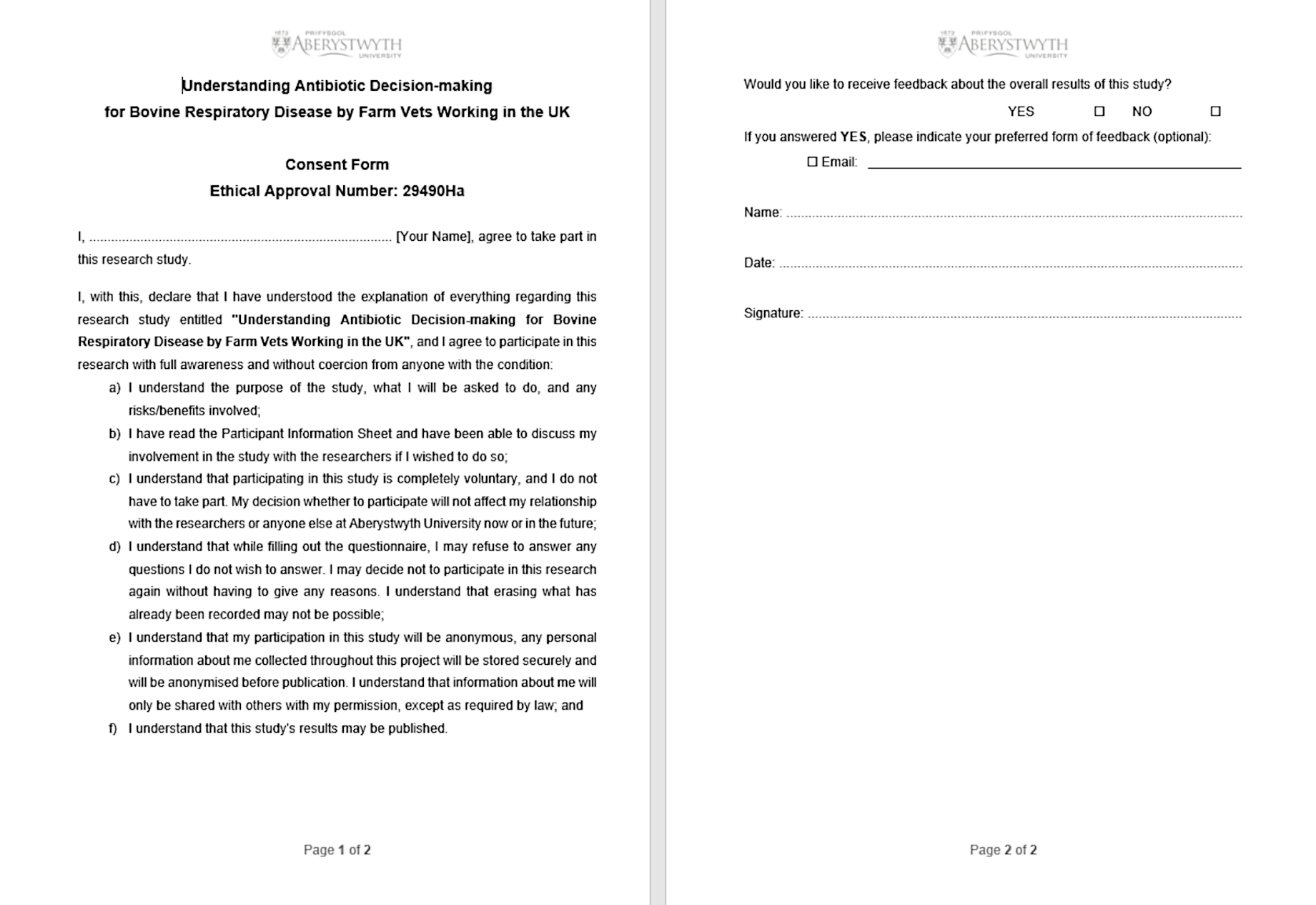


The survey as distributed.


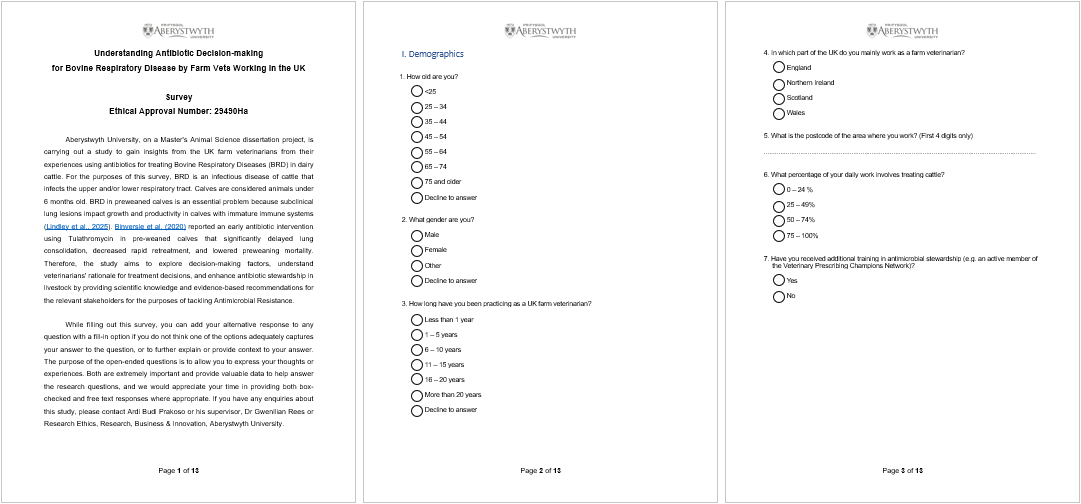


The survey as distributed (continued).


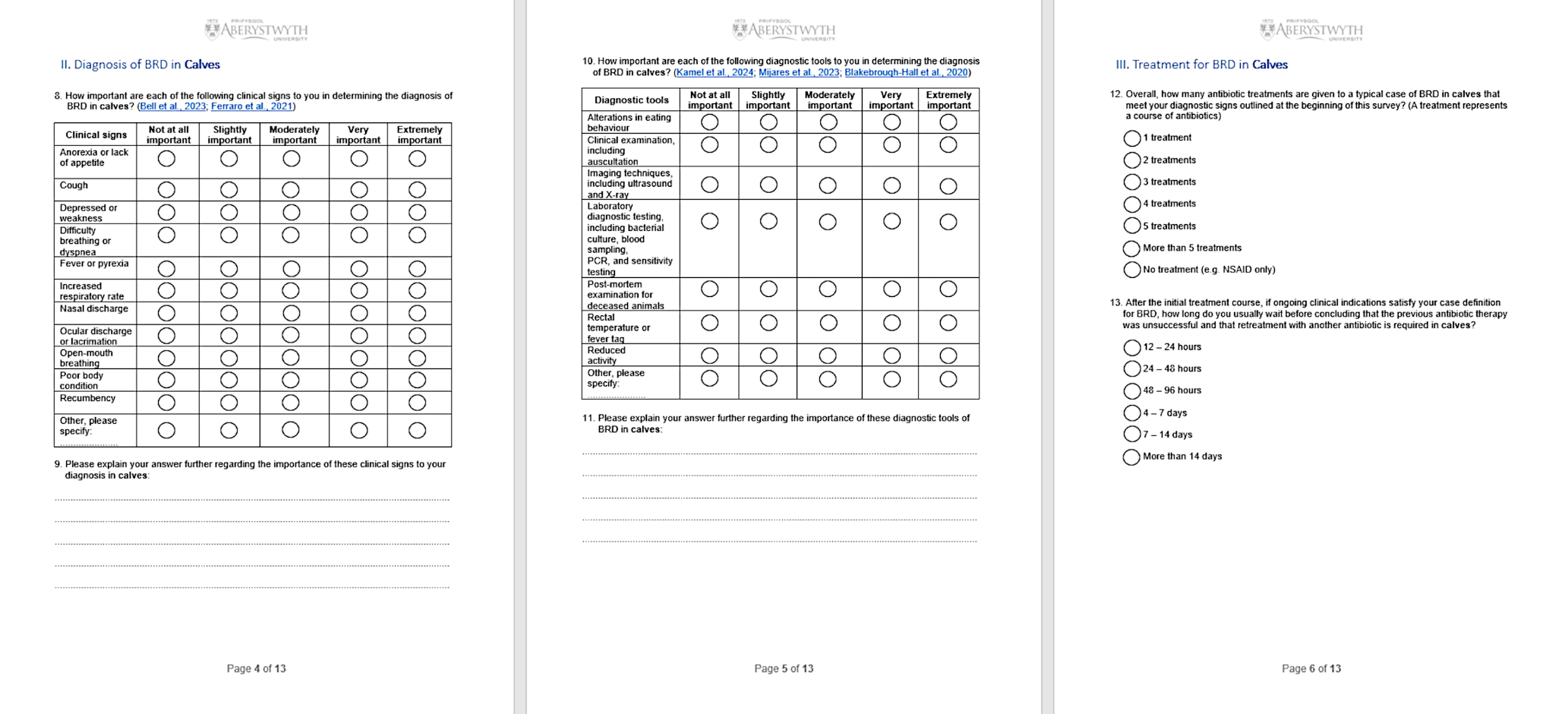


The survey as distributed (continued).


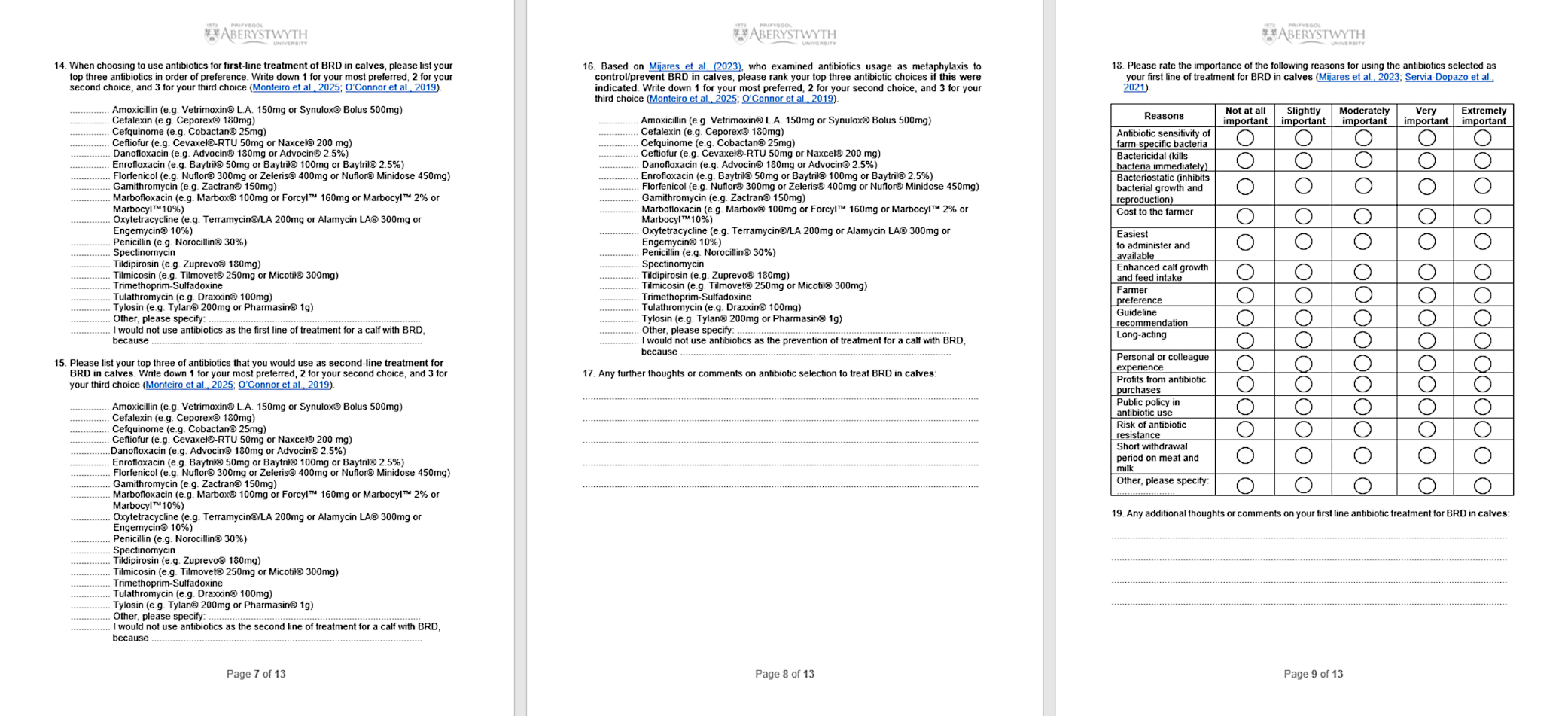


The survey as distributed (continued).


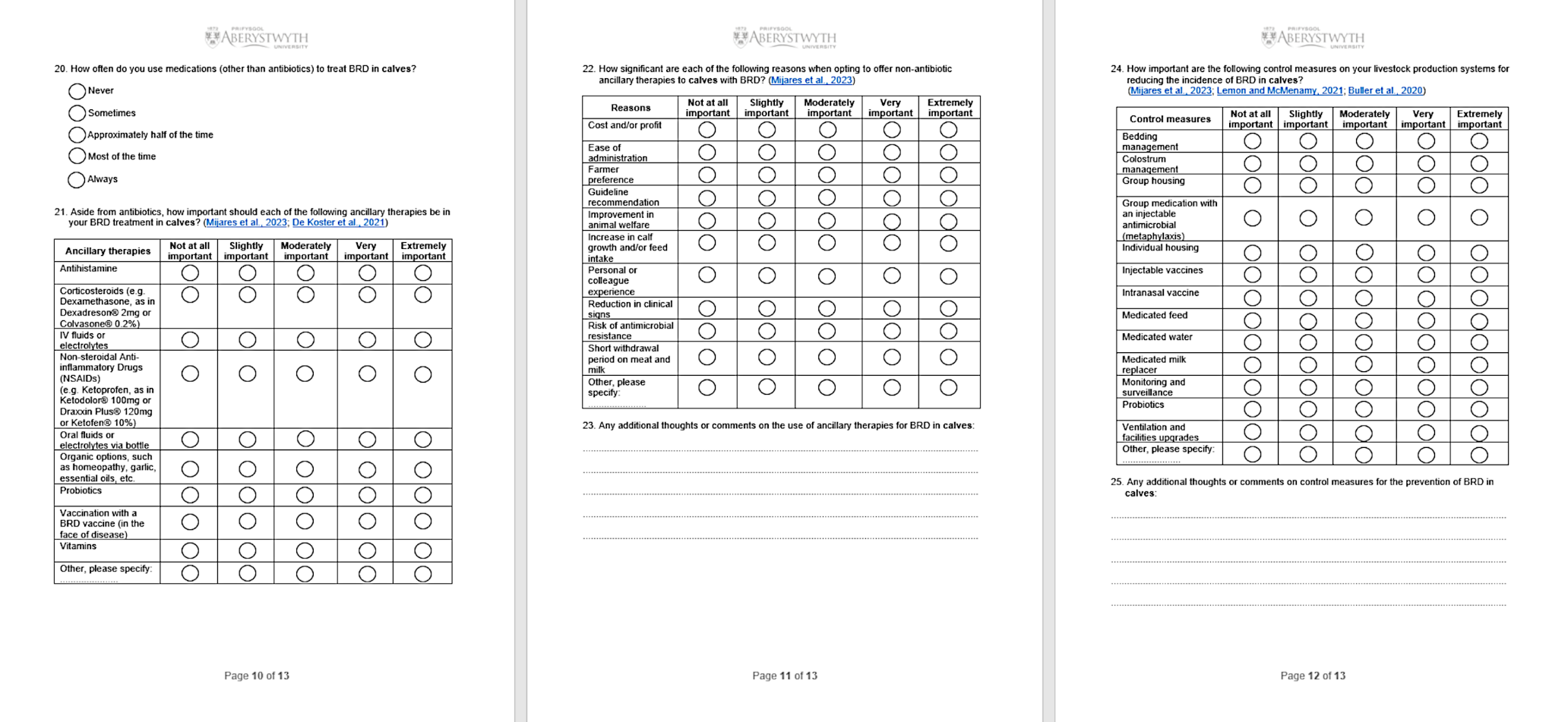


The survey as distributed (continued).


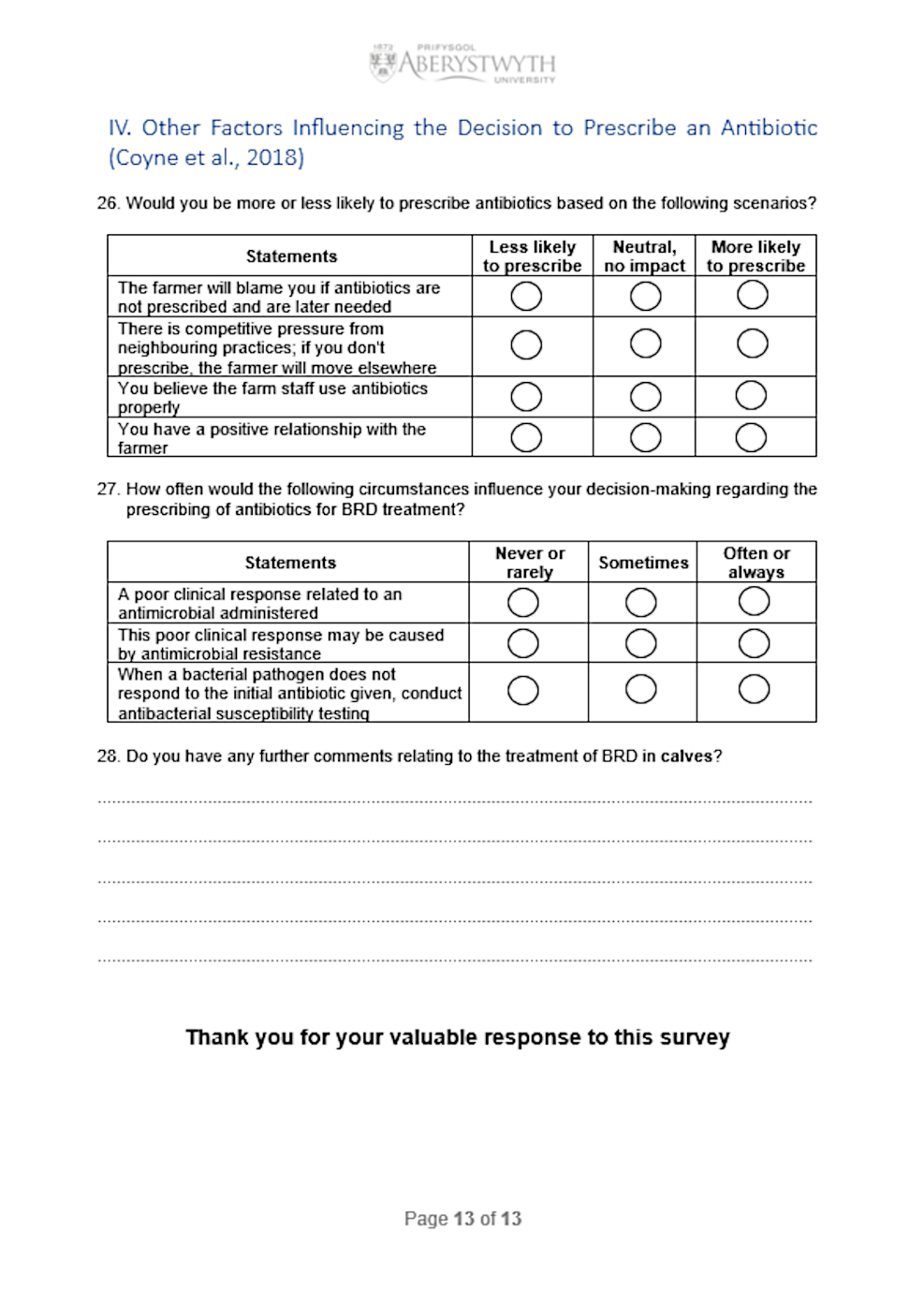


Supplemental Data Visualisations

This appendix contains a collection of individual graphical figures that support the study’s findings outlined in the Results section and provide additional graphs that capture the full survey’s responses.

| 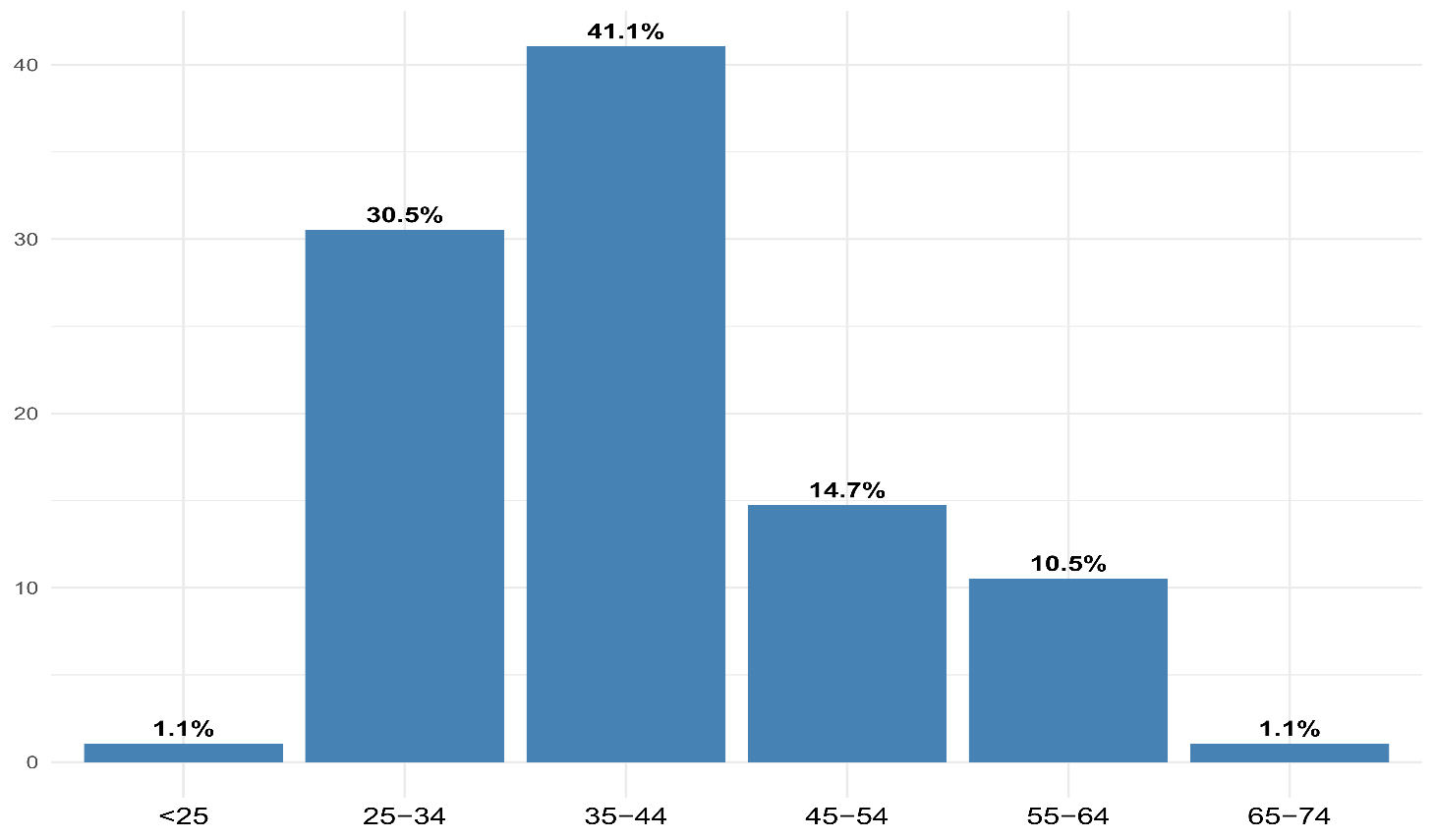 |
| --- |
|  |

**FIGURE 1**. Distribution of participants by age category (*n* = 95), shown as percentages.

Supplemental Data Visualisations (continued).


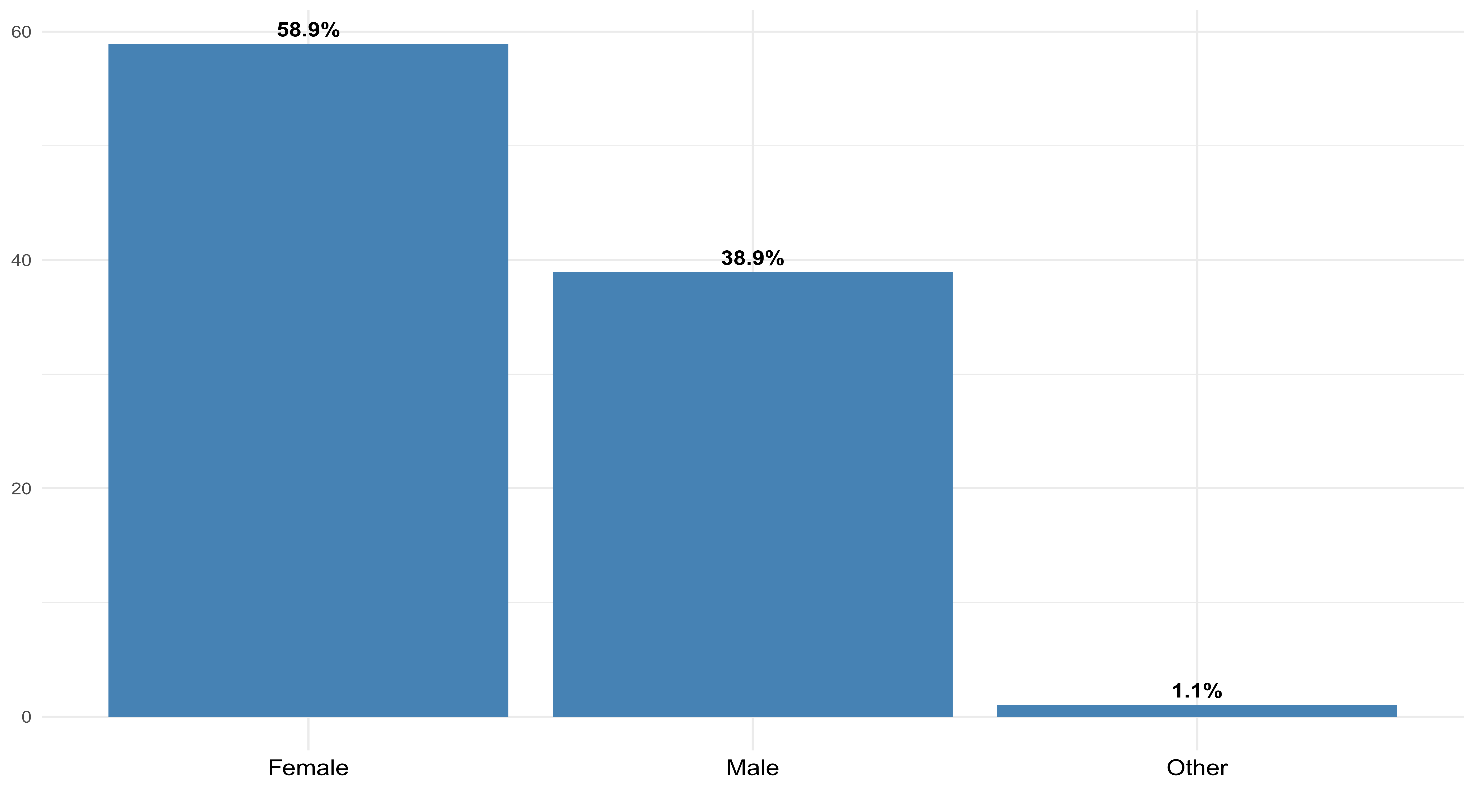


**FIGURE 2.** Distribution of participants by gender (*n* = 95), shown as percentages.

| 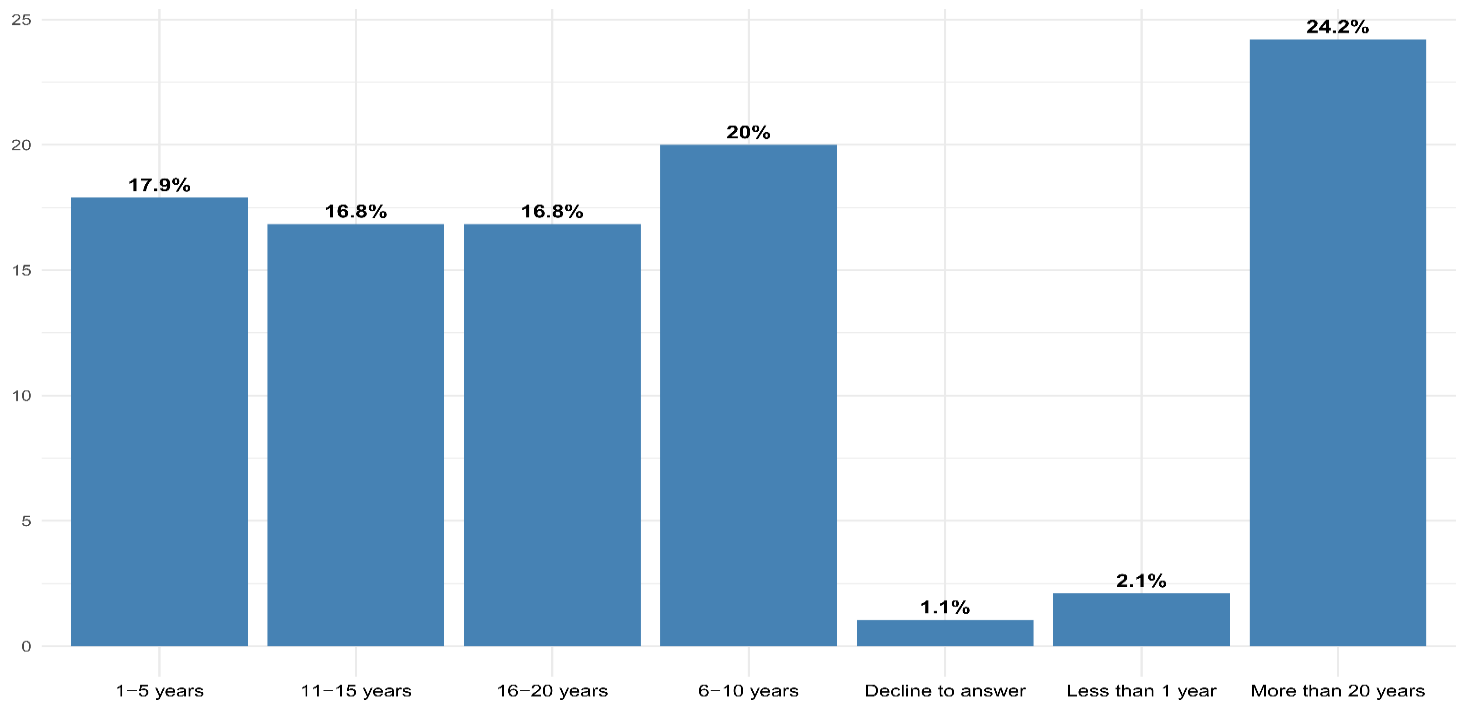 |
| --- |
|  |

**FIGURE 3.** Distribution of participants by duration of UK farm practice (*n* = 95), shown as percentages.

Supplemental Data Visualisations (continued).

| 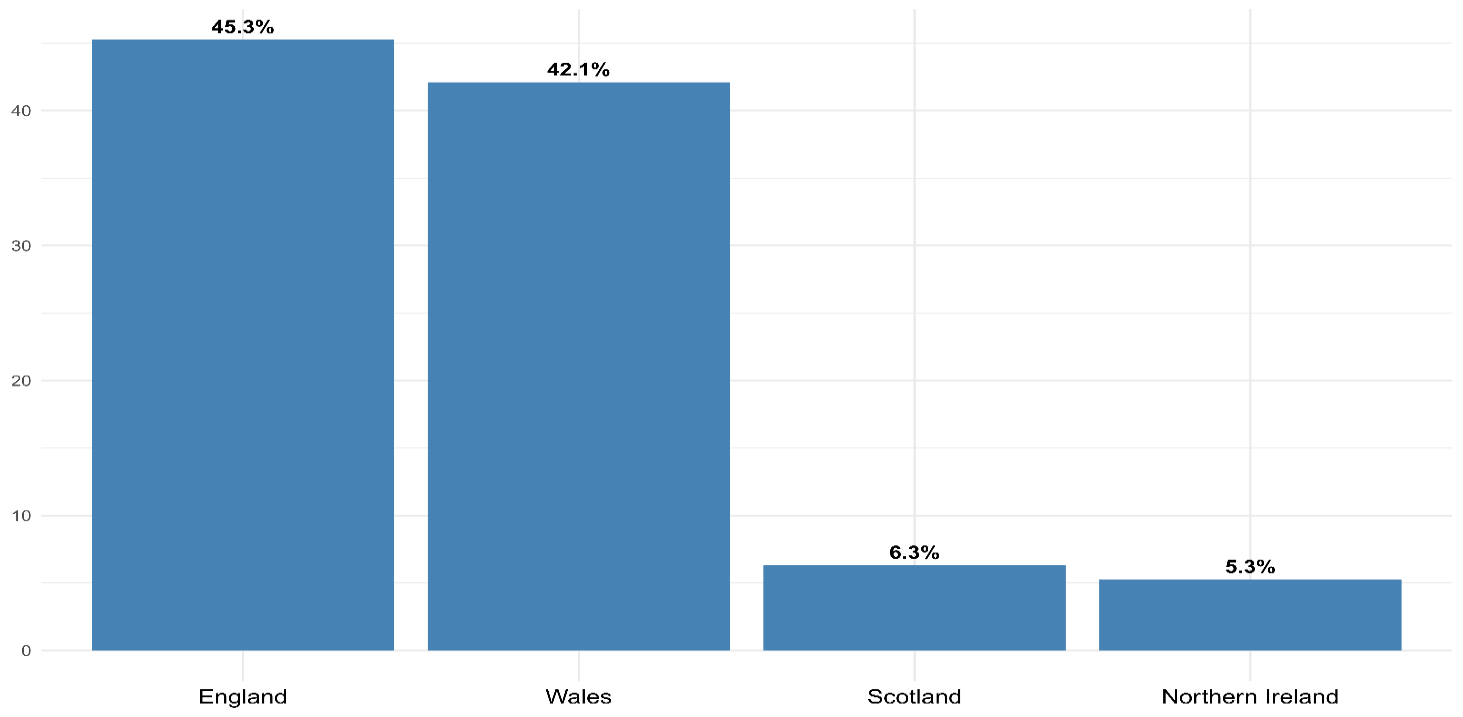 |
| --- |
|  |

**FIGURE 4.** Distribution of participants by primary work location (*n* = 95), shown as percentages.

| 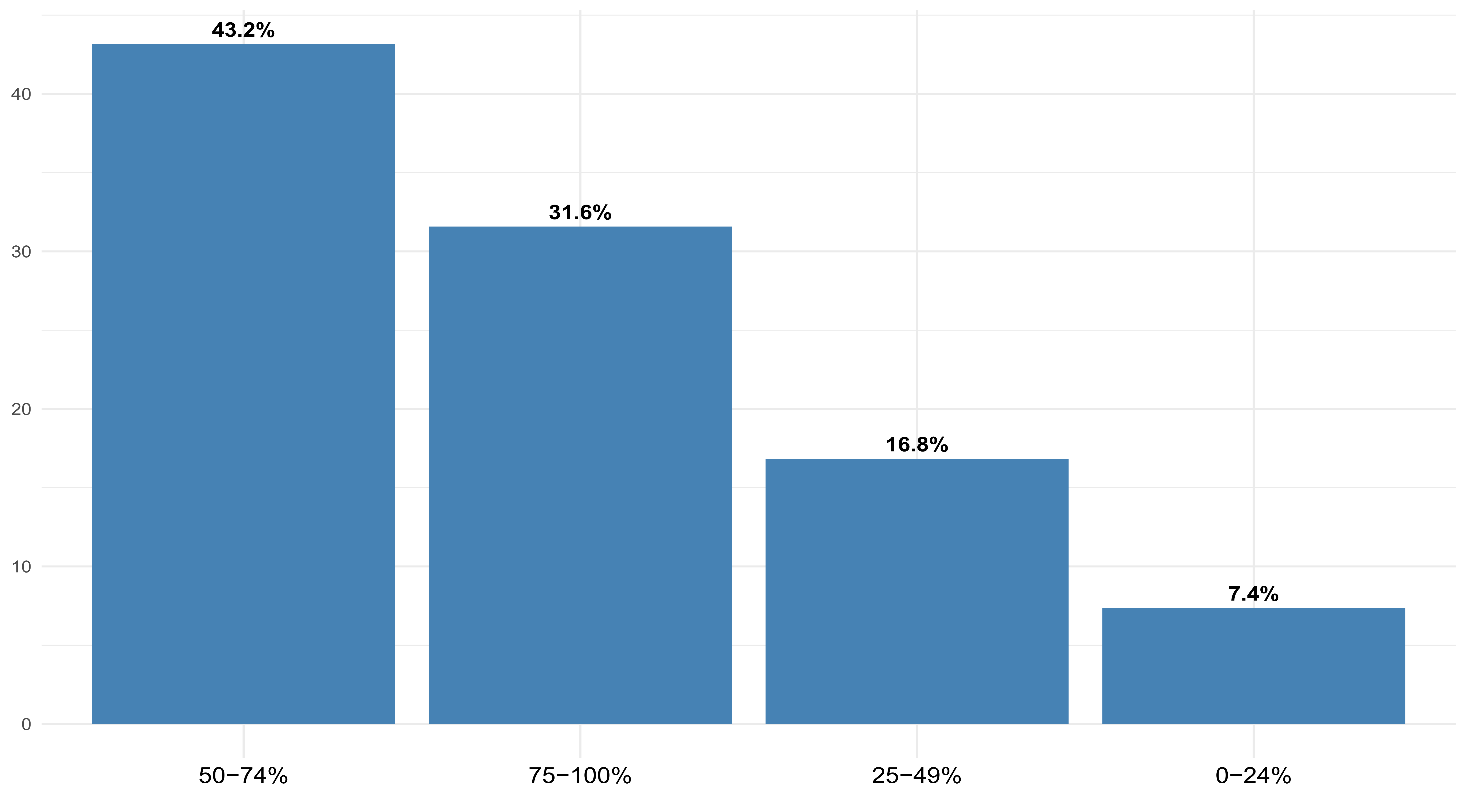 |
| --- |
|  |

**FIGURE 5.** Distribution of participants by the percentage of their daily work involving cattle treatment (*n* = 95), shown as percentages.

Supplemental Data Visualisations (continued).

| 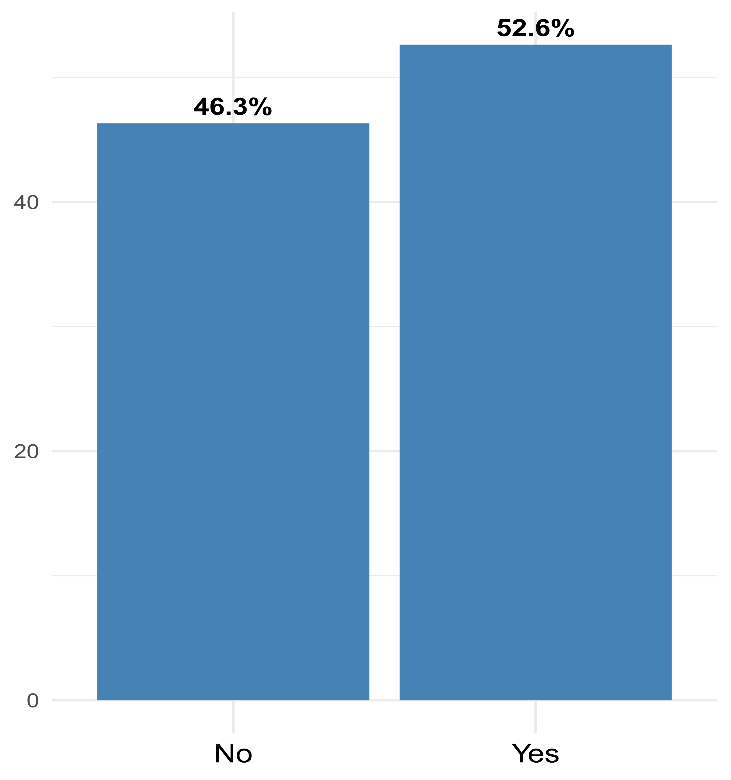 |
| --- |
|  |

**FIGURE 6.** Proportion of participants who had received formal training in antimicrobial stewardship (*n* = 95).


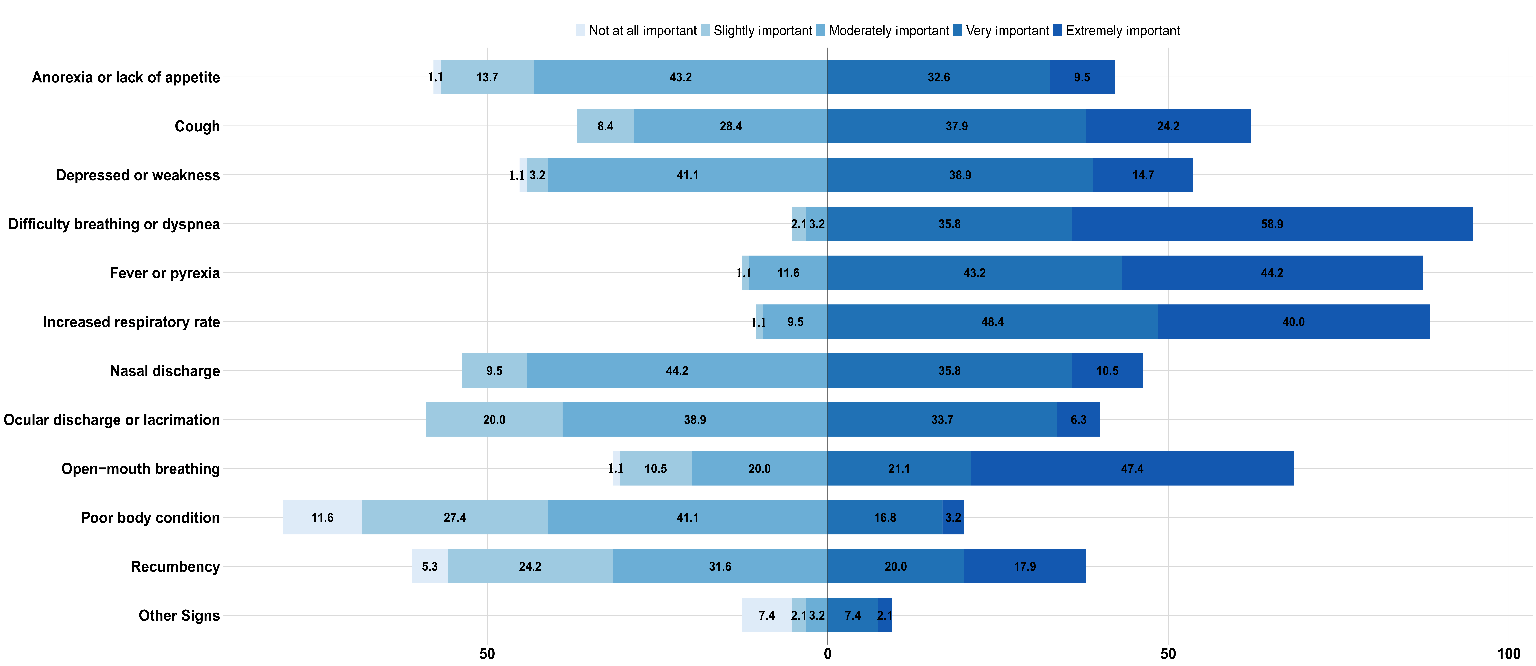


**FIGURE 7.** Percentage of participants rating the importance of each clinical sign from “Not at all” to “Extremely important” for diagnosing BRD in calves (*n* = 95).

Supplemental Data Visualisations (continued).

**
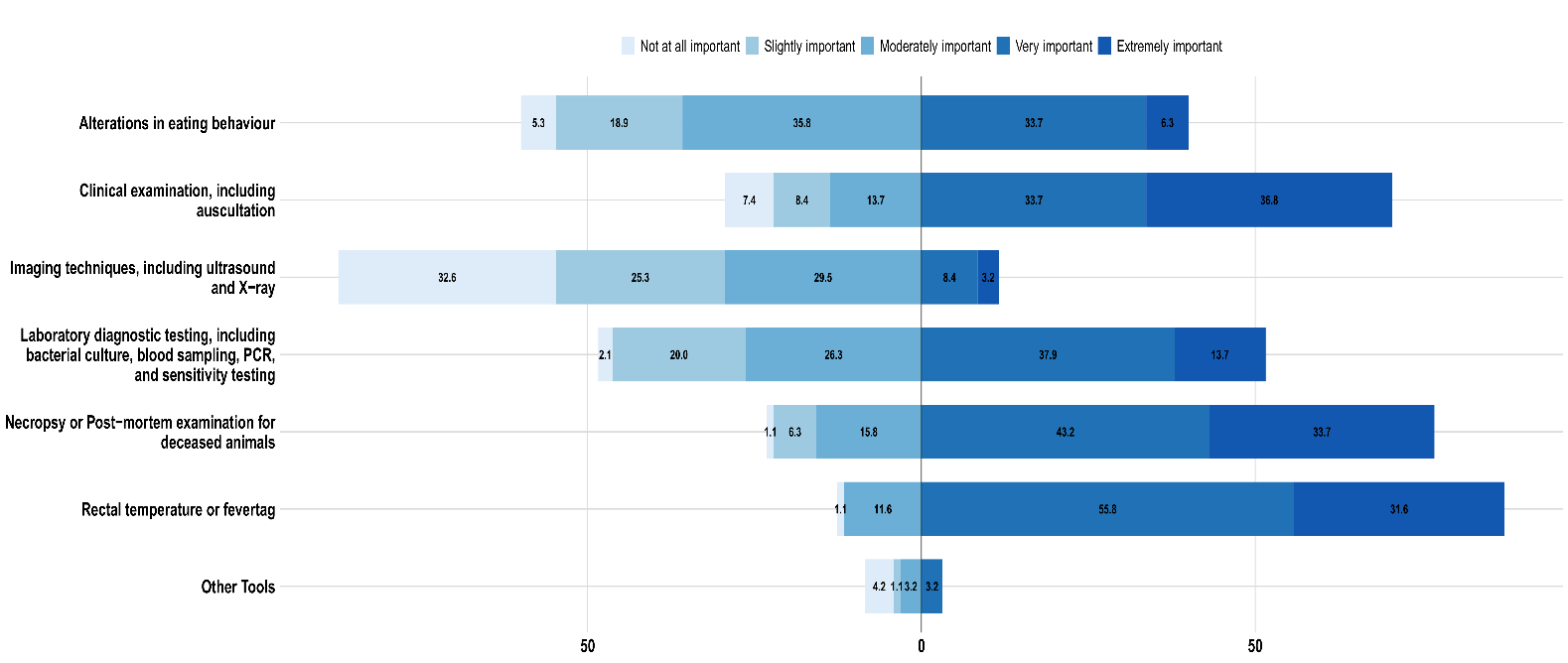
**

**FIGURE 8.** Percentage of participants rating the importance of each diagnostic tool from “Not at all” to “Extremely important” for BRD in calves (*n* = 95).

**
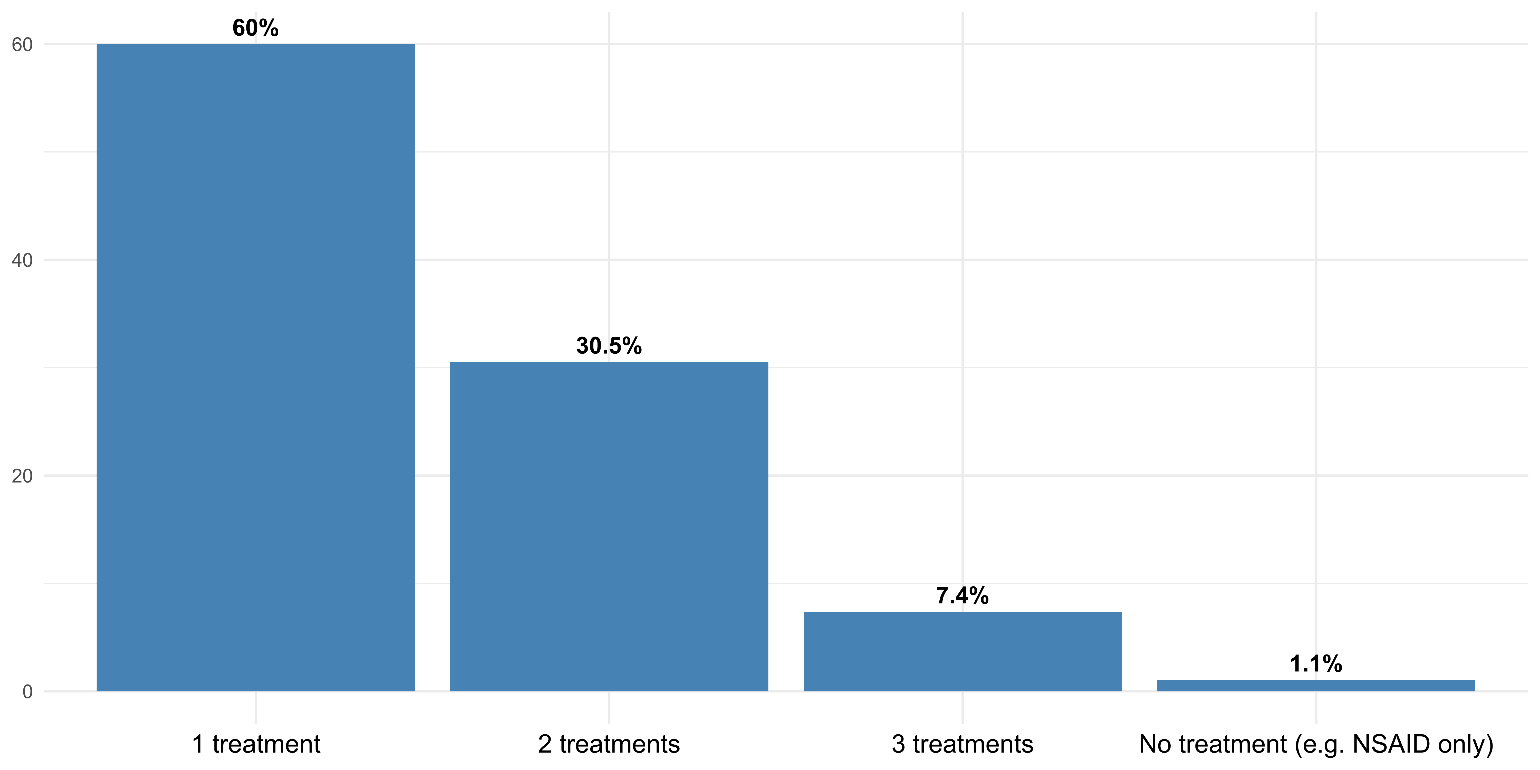
**

**FIGURE 9.** Percentage of participants (*n* = 95) by number of antibiotic treatments typically administered for calf BRD.

Supplemental Data Visualisations (continued).

| **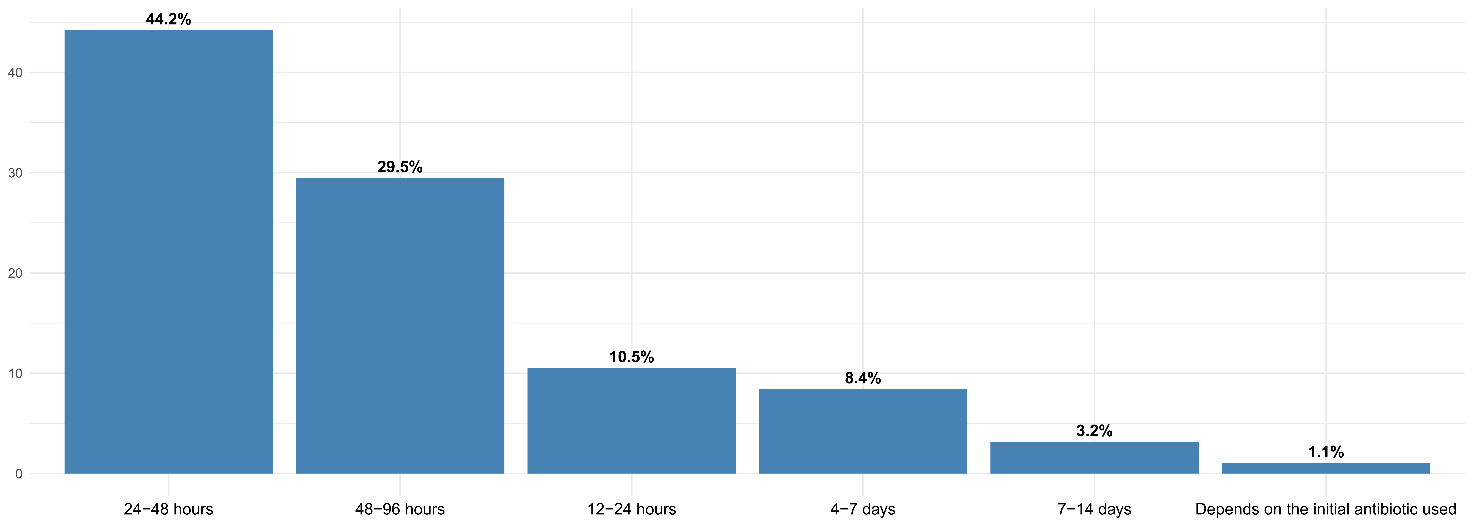** |
| --- |

**FIGURE 10**. Percentage of participants (*n* = 95) by waiting time before switching antibiotics after initial treatment failure for calf BRD.


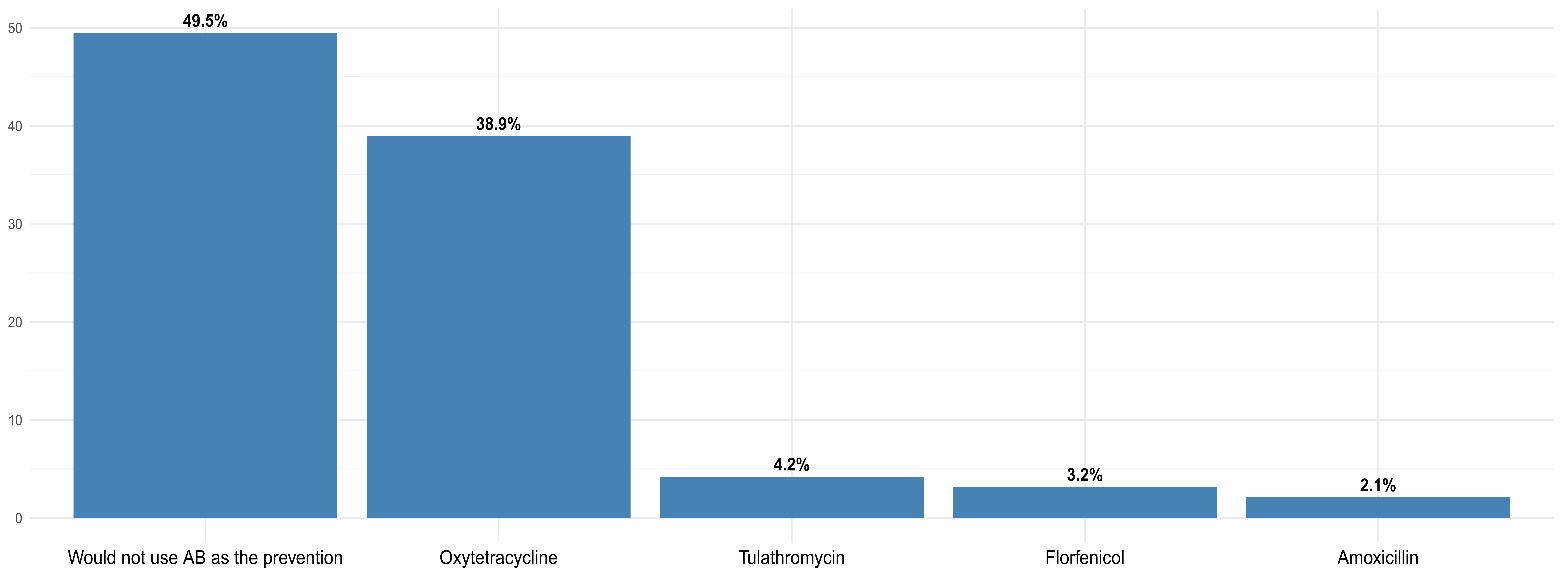


**FIGURE 11**. Percentage of participants’ (*n* = 95) first-choice antibiotic for BRD prevention in calves. AB = Antibiotics.

Supplemental Data Visualisations (continued).


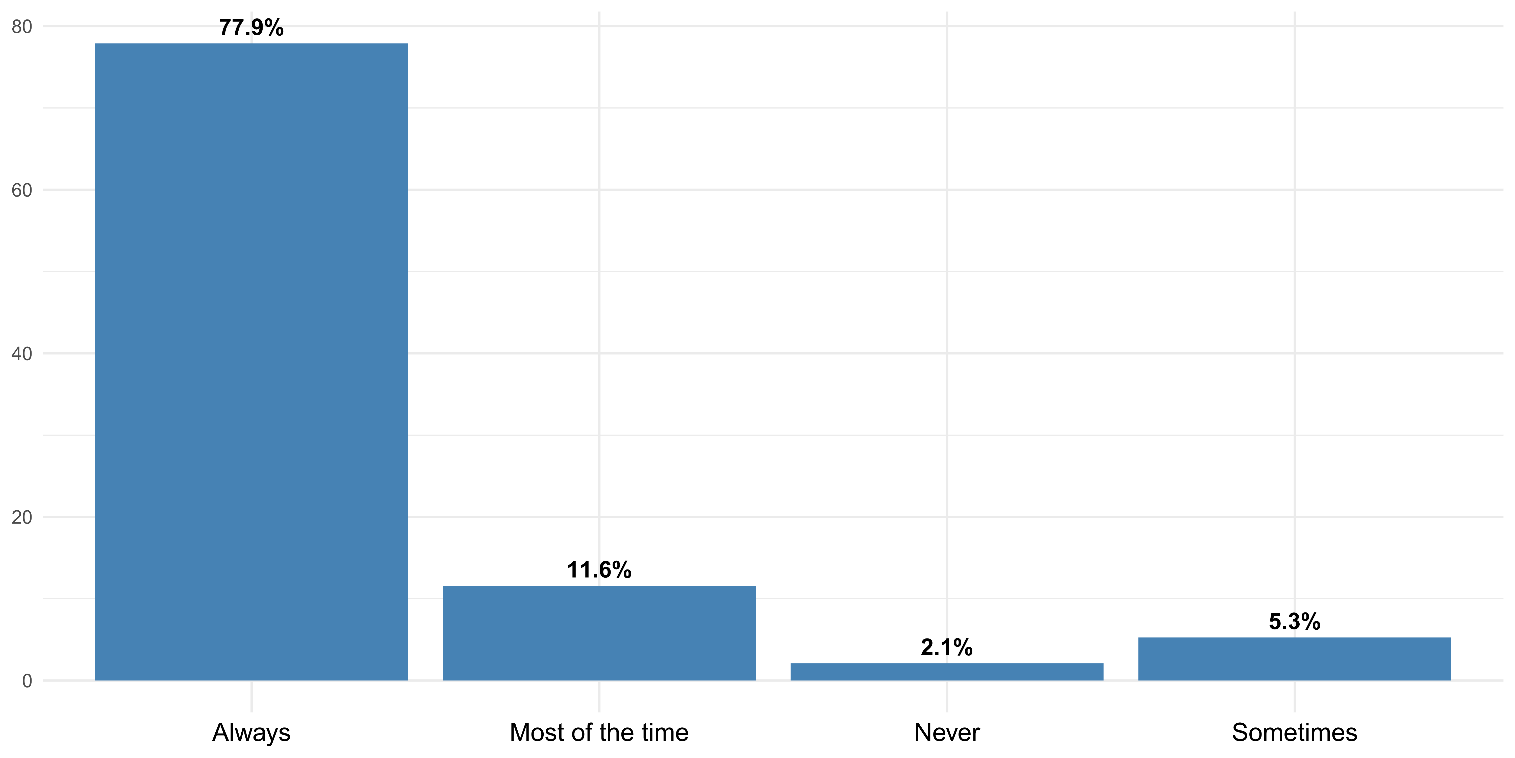


**FIGURE 12.** Distribution of participants by the frequency of using medications other than antibiotics for calf BRD treatment (*n* = 95), shown as percentages.

| 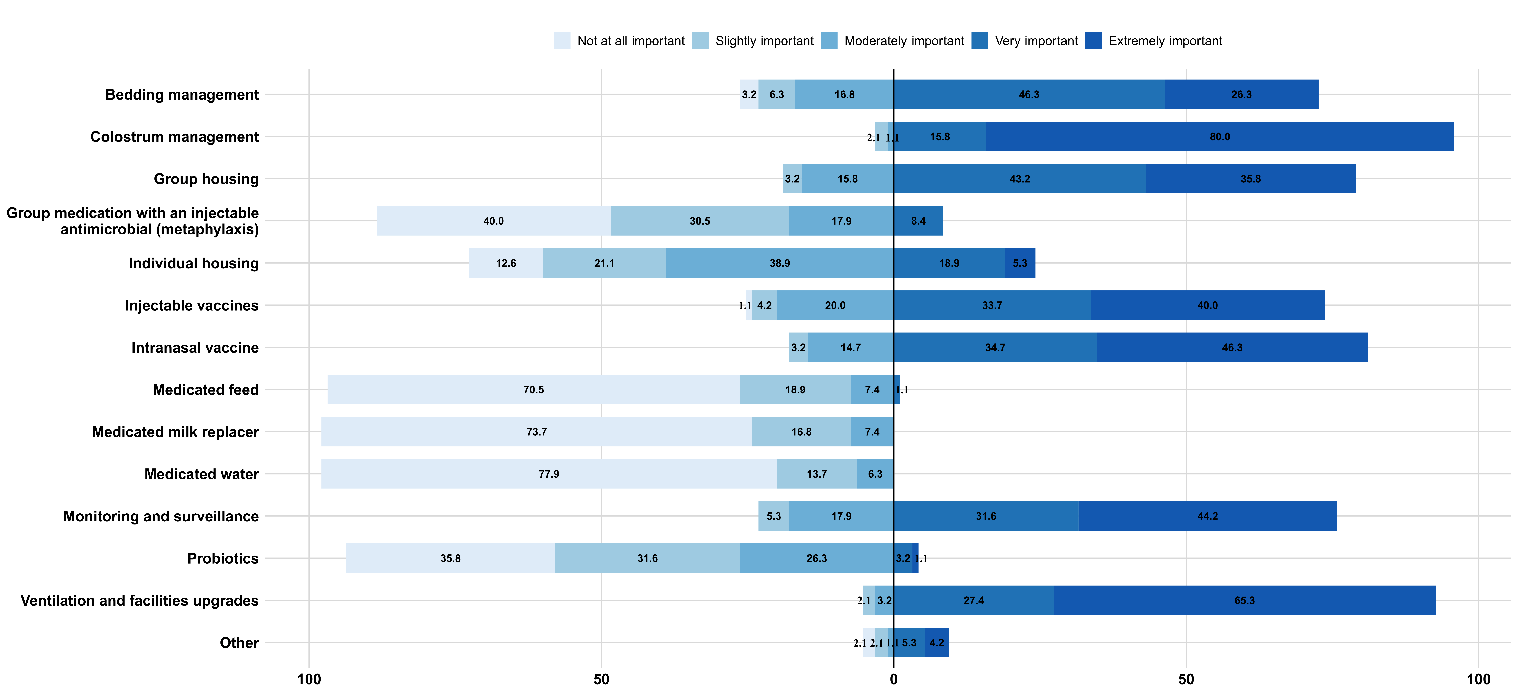 |
| --- |

**FIGURE 13.** Percentage of participants (*n* = 95) rating the importance of each control measure from “Not at all” to “Extremely important” for reducing calf BRD incidence.

Supplemental Data Visualisations (continued).

| 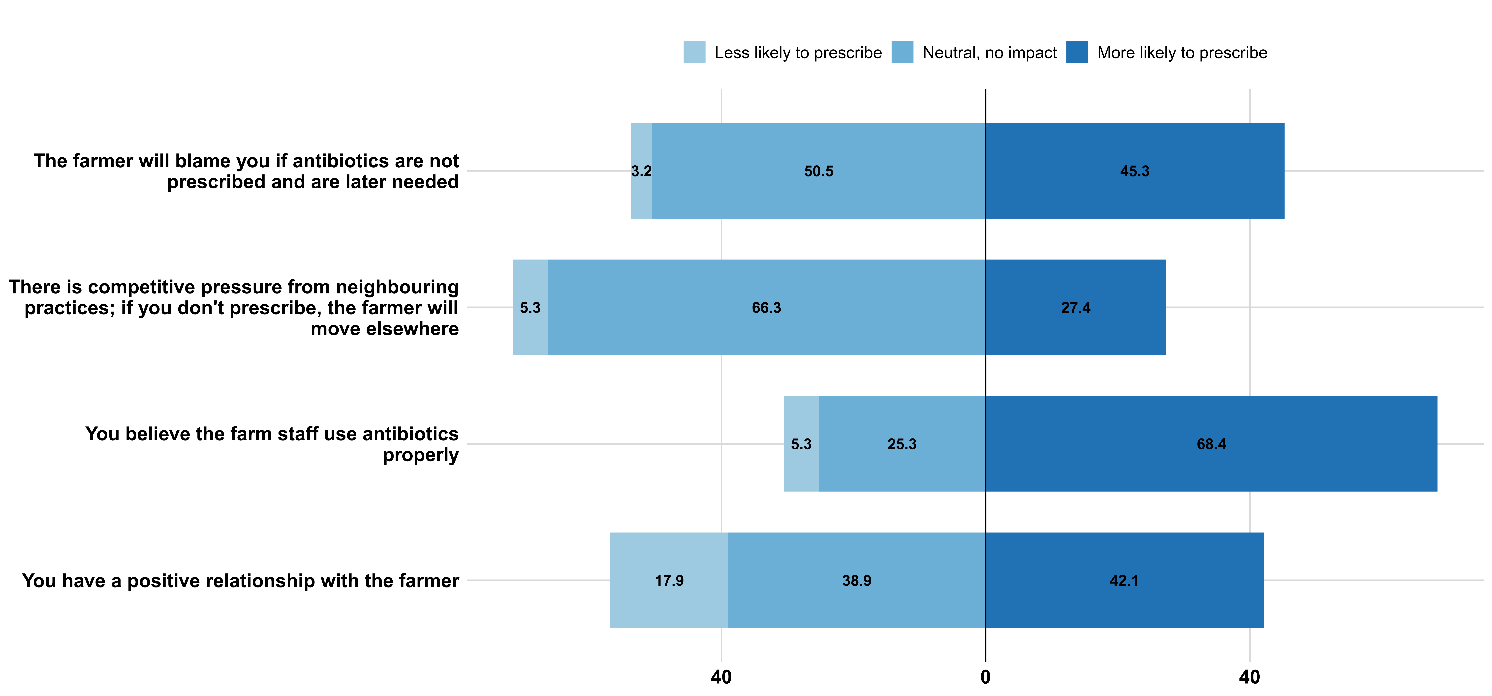 |
| --- |

**FIGURE 14.** Percentage of participants (*n* = 95) rating the likelihood of prescribing antibiotics from “Less likely” to “More likely” in various calf BRD scenarios.

| 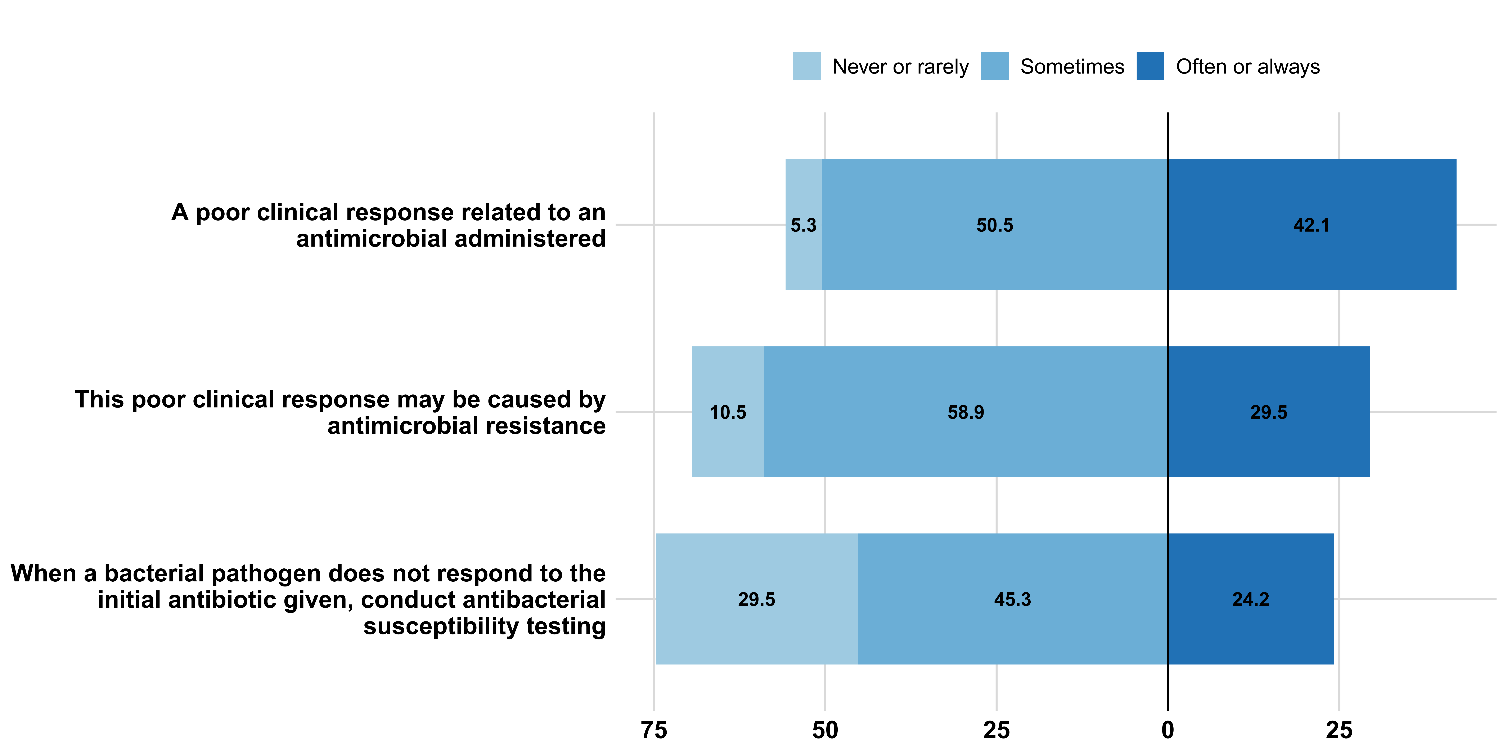 |
| --- |

**FIGURE 15.** Percentage of participants (*n* = 95) rating the influence of factors from “Never or rarely” to “Often or always” on antibiotic prescription for calf BRD treatment in each circumstance.
