# Supplementary material for: Understanding antibiotic decision-making for bovine respiratory disease: a survey of UK farm veterinarians
Source: Front Vet Sci. 2026 May 19;13:1791479. doi: 10.3389/fvets.2026.1791479 (PMC13225968; doi:10.3389/fvets.2026.1791479)
Supplement: Supplementary file 2 [file Supplementary_file_1.docx]

Supplementary Material

# 1 Supplementary Data

Further additional information and the full dataset of this research are provided in the Appendices. 

# 2 Appendices

The survey as distributed. 

The survey as distributed (continued). 

The survey as distributed (continued). 

The survey as distributed (continued). 

The survey as distributed (continued). 

		

Supplemental Data Visualisations

This appendix contains a collection of individual graphical figures that support the study’s findings outlined in the Results section and provide additional graphs that capture the full survey’s responses.

|  |
| --- |
|  |

**FIGURE S1**. Distribution of participants by age category (*n* = 95), shown as percentages. 

Supplemental Data Visualisations (continued).

**FIGURE S2.** Distribution of participants by gender (*n* = 95), shown as percentages. 

|  |
| --- |
|  |

**FIGURE S3.** Distribution of participants by duration of UK farm practice (*n* = 95), shown as percentages.

Supplemental Data Visualisations (continued).

|  |
| --- |
|  |

**FIGURE S4.** Distribution of participants by primary work location (*n* = 95), shown as percentages. 

|  |
| --- |
|  |

**FIGURE S5.** Distribution of participants by the percentage of their daily work involving cattle treatment (*n* = 95), shown as percentages.

Supplemental Data Visualisations (continued).

|  |
| --- |
|  |

**FIGURE S6.** Proportion of participants who had received formal training in antimicrobial stewardship (*n* = 95).

**FIGURE S7.** Percentage of participants rating the importance of each clinical sign from “Not at all” to “Extremely important” for diagnosing BRD in calves (*n* = 95). 

Supplemental Data Visualisations (continued).

**FIGURE S8.** Percentage of participants rating the importance of each diagnostic tool from “Not at all” to “Extremely important” for BRD in calves (*n* = 95).

**FIGURE S9.** Percentage of participants (*n* = 95) by number of antibiotic treatments typically administered for calf BRD.

Supplemental Data Visualisations (continued).

|  |
| --- |

**FIGURE S10**. Percentage of participants (*n* = 95) by waiting time before switching antibiotics after initial treatment failure for calf BRD.

**FIGURE S11**. Percentage of participants’ (*n* = 95) first-choice antibiotic for BRD prevention in calves. AB = Antibiotics.

Supplemental Data Visualisations (continued).

**FIGURE S12.** Distribution of participants by the frequency of using medications other than antibiotics for calf BRD treatment (*n* = 95), shown as percentages.

|  |
| --- |

**FIGURE S13.** Percentage of participants (*n* = 95) rating the importance of each control measure from “Not at all” to “Extremely important” for reducing calf BRD incidence. 

Supplemental Data Visualisations (continued). 

|  |
| --- |

**FIGURE S14.** Percentage of participants (*n* = 95) rating the likelihood of prescribing antibiotics from “Less likely” to “More likely” in various calf BRD scenarios. 

|  |
| --- |

**FIGURE S15.** Percentage of participants (*n* = 95) rating the influence of factors from “Never or rarely” to “Often or always” on antibiotic prescription for calf BRD treatment in each circumstance.
